# Interleukin-17 contributes to Ross River virus-induced arthritis and myositis

**DOI:** 10.1371/journal.ppat.1010185

**Published:** 2022-02-10

**Authors:** Helen Mostafavi, Kothila Tharmarajah, Jelena Vider, Nicholas P. West, Joseph R. Freitas, Barbara Cameron, Paul S. Foster, Linda P. Hueston, Andrew R. Lloyd, Suresh Mahalingam, Ali Zaid

**Affiliations:** 1 Emerging Viruses, Inflammation and Therapeutics Group, Menzies Health Institute Queensland, Griffith University, Gold Coast, QLD, Australia; 2 School of Medical Sciences, Griffith University, Gold Coast, QLD, Australia; 3 Global Virus Network (GVN) Centre of Excellence in Arboviruses, Gold Coast, QLD, Australia; 4 Mucosal Immunology Group, Menzies Health Institute Queensland, Griffith University, Gold Coast, QLD, Australia; 5 Viral immunology Systems Program, Kirby Institute, University of New South Wales, Kensington, Australia; 6 School of Biomedical Sciences, Faculty of Health Sciences and Hunter Medical Research Institute, University of Newcastle, Newcastle, NSW, Australia; 7 Arbovirus Emerging Diseases Unit, Centre for Infectious Diseases and Microbiology Laboratory Services, Pathology West—ICPMR Westmead, Australia; Washington University in Saint Louis, UNITED STATES

## Abstract

Arthritogenic alphaviruses are mosquito-borne viruses that are a major cause of infectious arthropathies worldwide, and recent outbreaks of chikungunya virus and Ross River virus (RRV) infections highlight the need for robust intervention strategies. Alphaviral arthritis can persist for months after the initial acute disease, and is mediated by cellular immune responses. A common strategy to limit inflammation and pathology is to dampen the overwhelming inflammatory responses by modulating proinflammatory cytokine pathways. Here, we investigate the contribution of interleukin-17 (IL-17), a cytokine involved in arthropathies such as rheumatoid arthritis, in the development RRV-induced arthritis and myositis. IL-17 was quantified in serum from RRV-infected patients, and mice were infected with RRV and joints and muscle tissues collected to analyse cellular infiltrates, tissue mRNA, cytokine expression, and joint and muscle histopathology. IL-17 expression was increased in musculoskeletal tissues and serum of RRV-infected mice and humans, respectively. IL-17–producing T cells and neutrophils contributed to the cellular infiltrate in the joint and muscle tissue during acute RRV disease in mice. Blockade of IL-17A/F using a monoclonal antibody (mAb) reduced disease severity in RRV-infected mice and led to decreased proinflammatory proteins, cellular infiltration in synovial tissues and cartilage damage, without affecting viral titers in inflamed tissues. IL-17A/F blockade triggered a shift in transcriptional profile of both leukocyte infiltrates and musculoskeletal stromal cells by downregulating proinflammatory genes. This study highlights a previously uncharacterized role for an effector cytokine in alphaviral pathology and points towards potential therapeutic benefit in targeting IL-17 to treat patients presenting with RRV-induced arthropathy.

## Author summary

Some viruses transmitted by mosquitoes cause painful and debilitating arthritis, which manifests both as an acute form shortly following infection, and a chronic form long after the initial symptoms have subsided. These viruses, termed arboviruses, are difficult to control and there are currently no specific treatments to alleviate the pain and loss of mobility. Arthritis caused by arboviruses shares similarities with a non-infectious, autoimmune form of arthritis called rheumatoid arthritis (RA). In RA, an immune molecule termed interleukin-17, or IL-17, has been shown to drive arthritis and treatments that target or block IL-17 are being developed to treat RA. Here, we asked whether arthritis caused by an arbovirus, Ross River virus (RRV), was also associated with elevated IL-17 in humans and mice. Disease severity in mice was associated with high IL-17 expression in the feet and muscle, and blocking IL-17 using an anti-IL-17 monoclonal antibody ameliorated disease in mice infected with RRV. Our study provides new information on a molecule that is implicated in arthritic inflammation, and could be targeted to treat disease caused by arthritogenic arboviruses.

## Introduction

Mosquito-borne viruses, or arboviruses, are a group of pathogens of major public health concern highlighted by several recent outbreaks of regional and global significance. Re-emerging arboviruses comprise two major families of RNA viruses, including flaviviruses (e.g. Zika) and alphaviruses (e.g. chikungunya, Ross River, Mayaro), each with their specific geographic distribution, mosquito vector specificity and disease aetiology. Arthritogenic alphaviruses, which include chikungunya (CHIKV), Ross River (RRV), Barmah Forest (BFV), Mayaro (MAYV) and o’nyong’nyong (ONNV) viruses cause severe joint and muscle inflammation leading to painful and debilitating arthritic disease [1–3]. While ~50–60% of patients recover from disease, some develop chronic arthralgia that can last months or years post-infection [4], however the mechanisms by which chronic illness develops following the initial acute infection are poorly understood.

Ross River virus (RRV) is an arthritogenic alphavirus endemic to Australia that is responsible for seasonal outbreaks that account for approximately 5000 cases each year, with recent, larger outbreaks affecting over 9000 people in 2015 [5], although it is also known to circulate in nearby southern Pacific islands [6]. RRV is primarily transmitted by *Aedes vigil*a*x* and *Culex annulirostris* mosquitoes, and is maintained via an enzootic cycle through a range of mammalian and marsupial hosts [6]. RRV disease (RRVD) usually manifests within the first 7–10 days post-infection and is characterised by a range of arthritic symptoms that also include fever, myalgia, lethargy and rash. Chronic arthralgia is common in RRVD patients and can persist for years, though the factors that trigger these persistent symptoms are not known. As the virus does not appear to persist in patient synovium beyond several weeks, it is not likely that the symptoms are virologically driven (as is also the case for CHIKV) [7,8]. It is possible that low grade chronic inflammation remains [9]. In the absence of vaccine, antivirals or targeted therapeutic solutions, RRV disease is generally treated with non-steroidal anti-inflammatory drugs (NSAIDs) or analgesics such as paracetamol, and recent clinical trials have shown some efficacy of pentosan polysulfate sodium (PPS), a sulphated polysaccharide, which is available as a prescription in Australia [10,11].

Innate immune responses have been shown to account for much of the inflammatory response that follows arthritogenic alphaviral inflammation, as they are required for efficient viral clearance [12,13]. However, T cell responses have also been shown to control viral replication and effect musculoskeletal inflammation following infection with arthritogenic alphaviruses [12,14–17], indicating that some effector T cell subsets may be driving, or contributing to inflammatory responses. Fingolimod (FTY720) and Abatacept (CTLA4-Ig), a suppressor of T cell lymphoid egress and T cell activation, respectively, reduced T cell accumulation, joint inflammation and disease in CHIKV-infected mice [18,19]. Some T cell subsets, including interleukin-17–producing T cells have been shown to play a role in rheumatoid arthritis (RA), and the interleukin-17 (IL-17) pathway is a current therapeutic target in the condition [20–23]. The interleukin 17 (IL-17) family consists of six structurally-related cytokines- IL-17A, IL-17B, IL-17C, IL-17D, IL-17E and IL-17F [24,25]. Although all IL-17 isoforms exhibit similar effects in target cells (such as recruiting neutrophils), they serve different functions (pathogenic and protective) in a tissue-specific manner [26]. The role of IL-17A in RA has been widely described, however less is known about IL-17B–F. IL-17B increases intestinal inflammation and neutrophil migration and promotes cancer cell proliferation [27,28], but can also be anti-inflammatory [29]. IL-17C promotes anti-microbial responses and maintains skin and intestine barriers [30,31], whereas IL-17D (the least studied) is elevated during viral infections and tumours [32,33]. IL-17E supports Th2 immune responses which stimulates the expansion of eosinophils, and has been shown to protect during parasitic helminth infections [34]. Of the IL-17 family, IL-17A and IL-17F are the most similar in their structure and function. Like IL-17A, IL-17F upregulates proinflammatory cytokines either directly, or together with TNF-α, IL-1β, G-CSF and matrix metalloproteases [35], and IL-17F was elevated in the synovium of RA patients [36].

IL-17 mediates its immune regulatory function by inducing the release of proinflammatory cytokines and chemokines in fibroblasts, osteoblasts, synoviocytes, macrophages, epithelial and endothelial cells [37,38]. These molecules then trigger the recruitment of innate immune cells such as macrophages and neutrophils to the site of infection to aid in clearing pathogens [39,40]. Cells that produce IL-17 include Th17 cells (CD4^+^IL-17^+^), Tc17 cells (CD8^+^ IL-17^+^), γδ T cells, natural killer (NK) cells, neutrophils and innate lymphoid cells (ILCs) [41]. While IL-17 exhibits a protective role in host defense against pathogenic infections in the epithelium and mucosal barrier [40,42,43], elevated levels of IL-17 have been detected in the synovium and synovial fluid of RA patients [44–49], and have been shown to exacerbate inflammation and bone damage in arthritis models such as RA [44,48–50]. Further, IL-17 was found to be a prognostic factor for poorer disease outcomes and severity [51,52], and promoted monocyte and neutrophil recruitment, in RA patients [53]. Of note, the attenuation of viral replication of Venezuelan equine encephalitis virus (VEEV), a non-arthritogenic alphavirus, positively correlated with increased IL-17 produced in human astrocytes [54]. Further, Th17 cell responses were modulated by IL-10 in mice infected with Sindbis virus (another alphavirus) [55].

Due to parallels between RA and alphavirus-induced arthritic disease (eg. chronic joint inflammation, increased cellular infiltrate in synovial tissue) [56,57], we asked whether IL-17 was likewise involved in driving musculoskeletal inflammation following infection with an arthritogenic alphavirus. Here, we used human sera and a mouse model of RRV-induced arthritis and myositis to understand the contribution of IL-17–producing T cell subsets, and examined the effects of IL-17 blockade *in vivo* on RRVD.

## Results

### RRV disease is associated with elevated IL-17 levels in patients and mice

**Fig 1:**
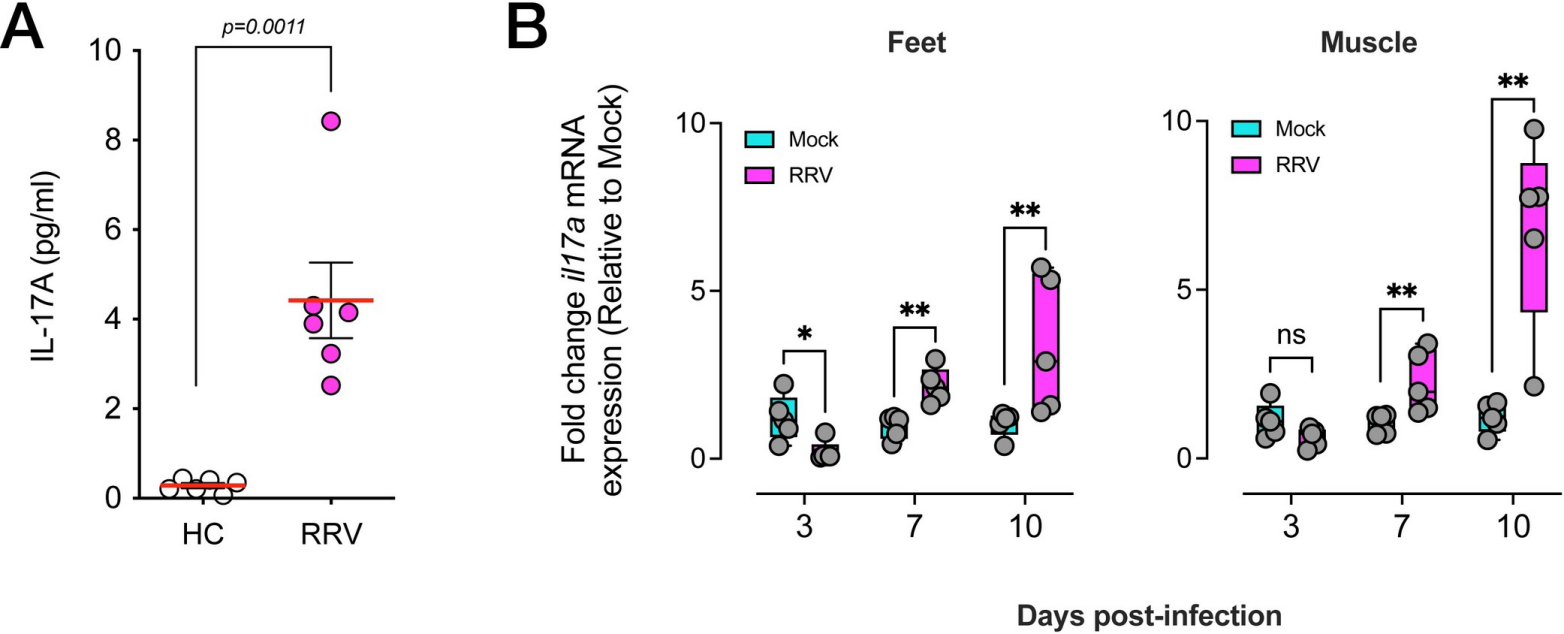
IL-17 is associated with RRV-induced arthritic disease in humans. A) Soluble IL-17A protein levels in the serum of healthy control (HC) and RRV-infected patients. Patient details described in Materials and Methods. Statistical analysis of differences between groups determined using a Mann-Whitney U test. **B)** mRNA expression of *Il17a* in the feet and muscle (quadriceps) of RRV-infected IL-17^GFP^ mice, at 3, 7 and 10 dpi. Expressed as fold-change relative to gene expression in mock-infected mice, and normalized to housekeeping gene expression, presented as mean +/- SEM. ** *p* < 0.01, Mann-Whitney U test. Data shows 3 independent experiments (n = 5 mice per group).

To determine whether IL-17 is expressed during acute RRV disease, we measured IL-17 protein levels in the serum of RRV-infected patients from the Dubbo Infection Outcomes Study (DIOS). In this cohort, levels of soluble IL-17A protein were significantly elevated during the acute phase of RRV disease (median of 4 days after onset) compared to the serum of healthy controls (Fig 1A). Next, we used our mouse model of RRV disease (RRVD) [58] to ask whether IL-17 expression was detectable in inflamed tissues during the acute phase of the disease. C57BL/6J mice were infected subcutaneously with RRV T_48_ and at 3, 7 and 10 days post-infection (dpi), feet and quadriceps muscle were collected, and total mRNA extracted. Quantitative real-time PCR showed that in both feet and muscle tissues, mRNA levels of IL-17A increased significantly during the course of RRV disease, and these peaked at 10 dpi, which corresponds to the peak of arthritic disease in mice (Fig 1B).

### RRV infection is associated with infiltration of T cells in the feet and muscle

**Fig 2:**
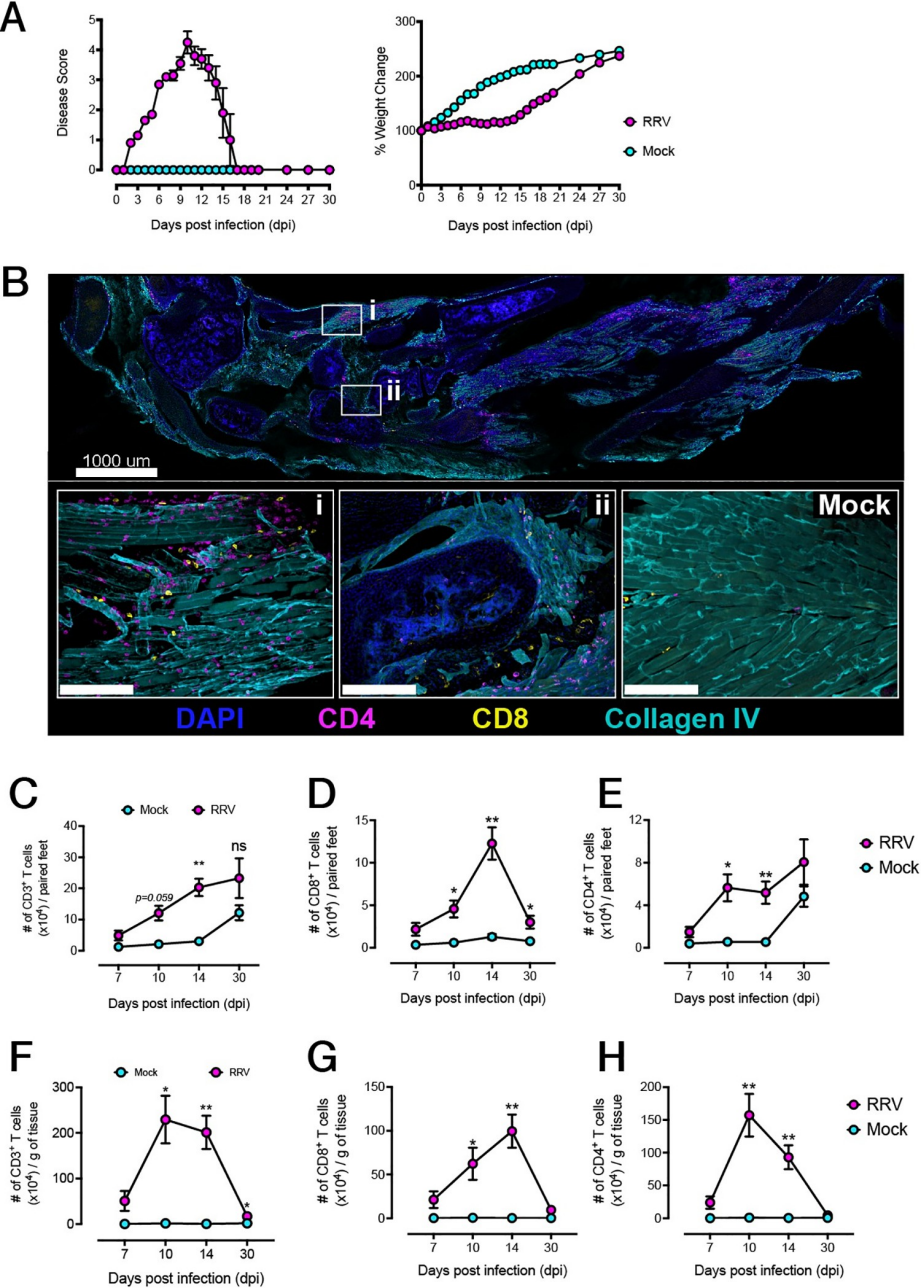
RRV-induced musculoskeletal inflammation is associated with T cell infiltration in the joints and muscle. **A)** Disease kinetics of IL-17^GFP^ mice infected with RRV_T48_. Mice were monitored for signs of disease and weight loss as described in Materials and Methods until 30 days post-infection (dpi). Data shown (mean +/- SEM) for 3 pooled independent experiments (n = 5 mice per group). **B)** Immunofluorescent microscopy of sagittal cryosection of the hindfoot of an RRV-infected IL-17^GFP^ mouse, at 10 dpi. Nuclei were stained with diaminido-2-phenylindol (DAPI), connective tissue was labelled using an anti-Type IV collagen antibody, and T cells labelled using anti-CD4 and anti-CD8 antibodies. Insets show the localization of infiltrating CD4^+^ and CD8^+^ T cells in the **(i)** interarticular muscle tissue and **(ii)** tenosynovium of RRV- and mock-infected mice. Data representative of 3 independent experiments (n = 4–5 mice per group). **C)** Quantification of CD3^+^ T cell infiltrates in the feet of mock-infected mice and RRV-infected IL-17^GFP^ mice at 7, 10, 14 and 30 dpi by flow cytometry. Data shown (mean +/- SEM) as number of LIVE CD45^+^CD3^+^ T cells per paired feet. **D)** Quantification of CD8^+^ and **E)** CD4^+^ T cell infiltrates in feet of mock-infected mice and RRV-infected IL-17^GFP^ mice at 7, 10, 14 and 30 dpi by flow cytometry. **F**) Quantification of CD3^+^ T cell infiltrates in the quadriceps muscle of mock-infected mice and RRV-infected IL-17^GFP^ mice at 7, 10, 14 and 30 dpi by flow cytometry. Data shown (mean +/- SEM) as number of LIVE CD45^+^CD3^+^ T cells per gram of muscle tissue. **G)** Quantification of CD8^+^ and **H)** CD4^+^ T cell infiltrates in muscle of mock-infected mice and RRV-IL-17^GFP^ infected mice at 7, 10, 14 and 30 dpi by flow cytometry. Data shown (mean +/- SEM) as number of LIVE CD45^+^CD3^+^CD8^+^ or LIVE CD45^+^CD3^+^CD4^+^ T cells per paired feet. Statistically significant differences between groups for each time point determined by Mann-Whitney U Test. *, *p* < 0.05; **, *p* < 0.01. Data representative of 3 independent experiments (n = 5 per group).

Next, we used the RRVD mouse model to investigate the spatio-temporal kinetics of leukocyte infiltration in the feet and muscle of RRV-infected mice [58]. In this model, RRV-infected mice display moderate to severe arthritic disease with an onset at approximately 7 dpi, and peak arthritic disease at approximately 10 dpi, during which mice experience slow weight gain, before subsiding until complete recovery (Fig 2A). Using wholemount immunostaining of foot sections from RRV-infected mice at 10 dpi, we investigated the localisation of T cells within the foot tissue architecture. Both CD4^+^ and CD8^+^ T cells were found primarily in the interarticular muscle that lines the synovium and connect metatarsal bones (Fig 2B; Inset *i*), as well as within the synovial tissue (Fig 2B; Inset *ii*), albeit to a lesser extent, whereas few T cells were observed in the feet of uninfected mice (Fig 2B).

We quantified leukocyte infiltrates in the feet using flow cytometry and observed that the total CD3^+^ T cell numbers in the feet increased steadily throughout disease progression, between days 7 and 14 (Fig 2C). The number of CD8^+^ T cells in the feet was significantly higher compared to uninfected mice at 10 dpi, and was highest at 14 dpi (Fig 2D), with a small, contracted population remaining by 30 dpi. CD4^+^ T cell numbers in the feet were likewise significantly higher compared to uninfected mice at 10 and 14 dpi (Fig 2E). Unlike CD8^+^ T cells, CD4^+^ T cells numbers did not decline by 30 dpi in the feet, and interestingly, uninfected feet also showed an increase in the number of CD4^+^ T cells. In the muscle, CD3^+^ T cell infiltrated the tissue in large numbers from 10 to 14 dpi, with numbers declining by 30 dpi (Fig 2F); this correlated with a peak in CD8^+^ T cells infiltration at 14 dpi (Fig 2G) and CD4^+^ T cell infiltration at 10 dpi (Fig 2H). The proportions of infiltrating CD3^+^ T cells (of total CD45^+^ non-myeloid Lin^-^ cells) increased in the feet and muscle over the course of RRV infection (S1A and S1B Fig). While the percentage of CD4^+^ peaked at 10 dpi in both tissues, that of CD8^+^ T cells was highest at 14 dpi (S1A and S1B Fig). By 30 dpi, the number of T cells in the muscle had substantially contracted to pre-onset levels, however a number of CD4^+^ and CD8^+^ T cells appeared to be maintained in the feet. We investigated the possibility that these may be tissue-resident T cells (T_RM_) that remain in the tissues after inflammation and viral clearance. Neither subset displayed the typical phenotype observed in T_RM_ (S2 Fig), where upregulation of CD103 and downregulation of KLRG1 denotes both a tissue-resident and suppressed T cell senescence program, respectively [59,60].

### IL-17–expressing T cells infiltrate the feet and muscle during acute RRV infection

**Fig 3:**
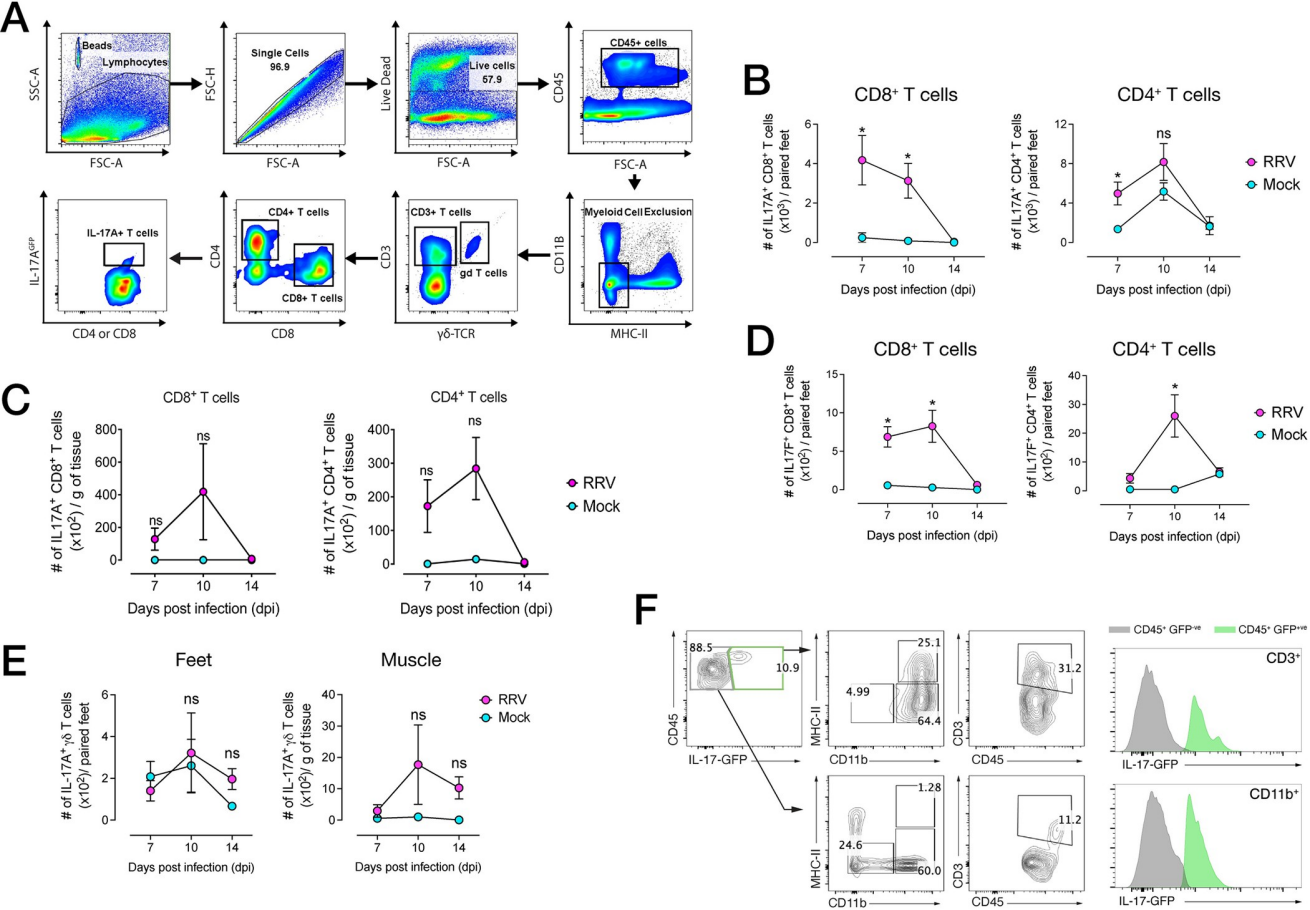
IL-17–producing cells infiltrate musculoskeletal tissues in RRV disease. **A)** Gating strategy used to quantify infiltrating cells in the feet in RRV-infected IL-17^GFP^ mice. Live singlet cells were gated for CD45^+^ and subsequently excluded from the CD11b^+^ MHC-II^+^ myeloid lineage (Lin) gate. CD3^+^ cell and subsequent CD4^+^ and CD8^+^ cell populations gated on IL-17A^+^ (or IL-17F^+^), and γδTCR-positive T cells (γδ T cells) were gated from the CD3^+^γδTCR^+^ cell population. **B**) IL-17-expressing CD8^+^ and CD4^+^ T cells in the feet and **C)** quadriceps muscle of RRV-infected IL-17^GFP^ mice at 7, 10, 14 and 30 dpi. Data shown (mean +/- SEM) as number of LIVE CD45^+^Lin^-^CD3^+^CD8^+^IL-17A^GFP+^ or LIVE CD45^+^Lin^-^CD3^+^CD4^+^IL-17A^GFP+^ T cells, per paired feet or per gram of muscle tissue. **D)** IL-17F^+^ CD8^+^ and CD4^+^ T cells in the feet of RRV-infected IL-17^GFP^ mice at 7, 10 and 14 dpi. Data shown (mean +/- SEM) as number of LIVE CD45^+^ Lin^-^CD3^+^CD8^+^IL-17F^+^ or LIVE CD45^+^ Lin^-^CD3^+^CD4^+^IL-17F^+^ T cells, per paired feet. **E)** IL-17A^+^ γδ T cells in the feet (left) and quadriceps muscle (right) of RRV-infected IL-17^GFP^ mice at 7, 10 and 14 dpi. Data shown (mean +/- SEM) as number of LIVE CD45^+^ Lin^-^CD3^+^CD4^-^CD8^-^ γδTCR^+^ T cells, per paired feet, or per gram of muscle tissue. IL-17F expression were assessed by intracellular cytokine staining. Data in **B-E** representative of 3 independent experiments (n = 5 mice per group) and statistically significant differences between groups for each time point determined by Mann-Whitney U Test. *, *p* < 0.05; **, *p* < 0.01; ns: not significant. **F)** Flow cytometry gating strategy for IL-17^GFP+^ CD45^+^ cells and corresponding histogram (normalized counts from concatenated samples) for IL-17^GFP^ reporter expression in CD3^+^ T cells and CD11b^+^ myeloid cells in the joints of RRV-infected mice, at 10 dpi. Populations gated from GFP^-ve^ (grey histogram) and GFP^+ve^ (green histogram). Mean population frequencies shown in gates. Data representative of 3 independent experiments (n = 5 mice per group).

We next used flow cytometry using RRV-infected IL-17^GFP^ mice to determine whether these foot-infiltrating, synovial T cells expressed IL-17 (Fig 3A), and found that both CD8^+^ and CD4^+^ T cells expressed IL-17A, primarily at 7 and 10 dpi, with both numbers declining by 14 dpi, when mice begin to recover (Fig 3B). In the muscle, a peak in the number of IL-17A^+^ CD8^+^ and CD4^+^ T cells was likewise observed between 7 and 10 dpi, though these increases were not statistically significant (Fig 3C). Overall, the proportions of IL-17A^+^ T cells in the feet and muscle (S1C and S1D Fig) were low compared to non-IL-17–expressing T cells, ranging between 2–5% of their parent CD4^+^ or CD8^+^ populations. Because the IL-17 receptor complex can be engaged both by IL-17A and IL-17F isoforms [24], we asked whether T cells found in the inflamed feet of RRV-infected mice produced IL-17F. IL-17F^+^ CD8^+^ and CD4^+^ T cells were found in the feet at 7 and 10 dpi, with IL-17F^+^ CD4^+^ T cells most abundant at 10 dpi (Fig 3D). By 14 dpi, the number of IL-17F^+^ T cells in the feet was substantially reduced (Fig 3D). T cells expressing the γδTCR^+^, or γδ T cells, have also been shown to produce IL-17A in arthritic disease (RA) in mice and humans [61–63]. In our model, the number (Fig 3E) and proportions (S1E Fig) of IL-17A^+^ γδ T cells in the feet was negligible compared to that of αβTCR^+^ T cells, whereas in the muscle, a small increase in overall numbers was observed, but was not statistically significant. Interestingly, we found that both infiltrating CD3^+^ (T cells) and CD11b^+^ (myeloid cells) subsets expressed GFP in inflamed feet (10 dpi), indicating that IL-17 expression was not confined to tissue-infiltrating T cells (Fig 3F).

**Fig 4:**
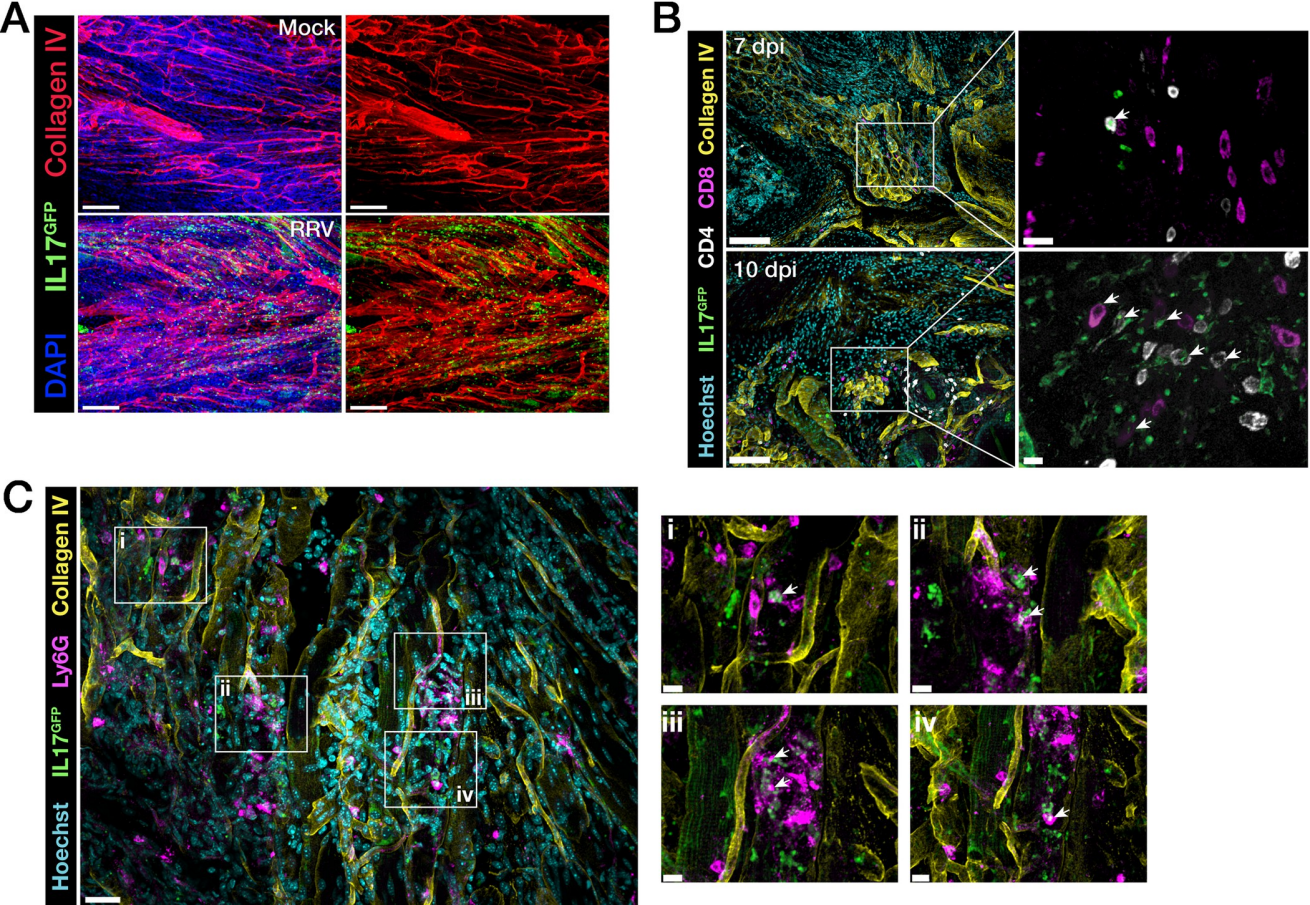
IL-17–expressing cells infiltrate joint tissues during RRV disease. **A)** Confocal microscopy of immunofluorescent staining of optically cleared 150μm−thick vibratome sections of feet from mock-infected or RRV-infected IL-17^GFP^ mice, at 10 dpi. Scale bars = 50μm. Wholemount sections were stained for Type IV Collagen (red) and nuclei labelled with DAPI (blue). **B)** Immunofluorescent staining of CD4^+^, CD8^+^ T cells and Type IV Collagen in foot cryosections from mock-infected or RRV-infected IL-17^GFP^ mice at 7 and 10 dpi. Scale bars = 100μm (left panels) and 15μm (right panels). **C)** Immunofluorescent staining of Ly6G^+^ neutrophils and Type IV Collagen in foot cryosections RRV-infected IL-17^GFP^ mice, at 10 dpi. Arrows indicate GFP^+ve^ cells. Nuclei were labelled with Hoechst 33258. Scale bars = 30μm (left panel) and 10μm (insets). Images acquired as z-stacks and shown as maximum intensity projections. Data representative of 3 independent experiments (n = 5 mice per group).

**Fig 5:**
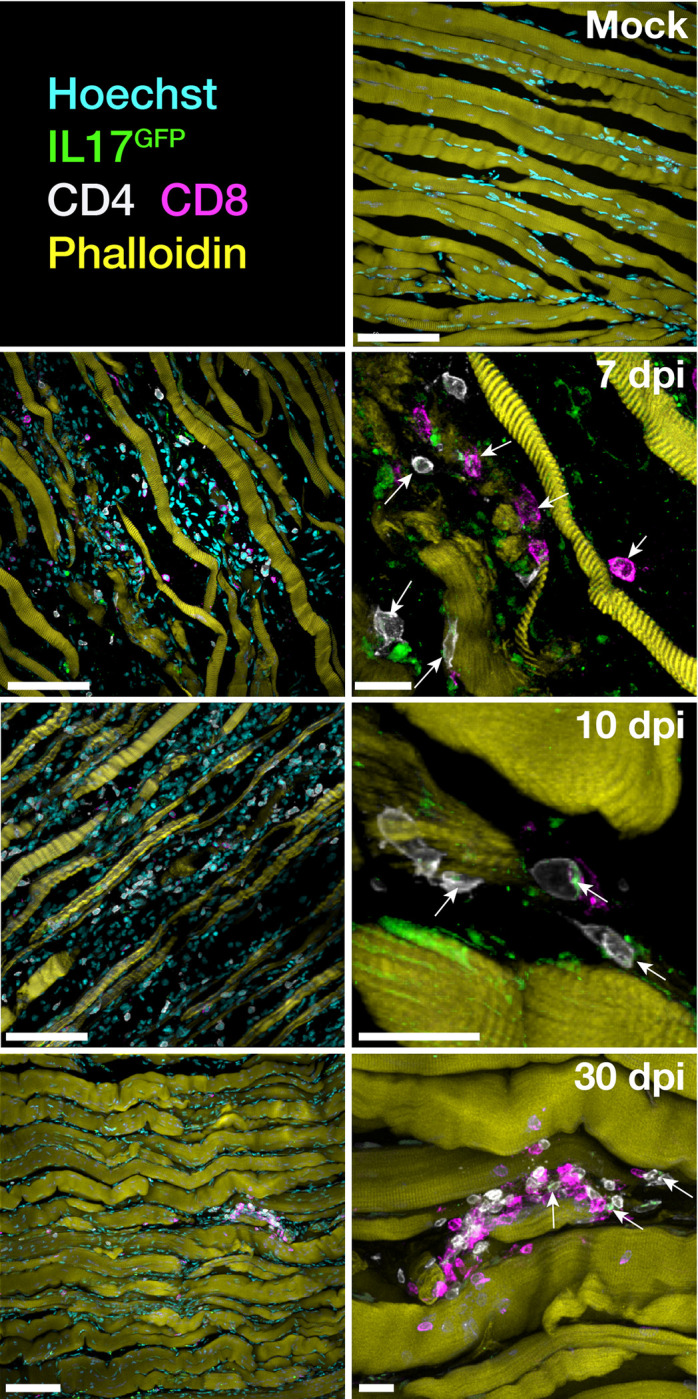
IL-17–producing cells infiltrate skeletal muscle during RRV disease. Immunofluorescent staining of quadriceps muscle cryosections of mock-infected or RRV-infected IL-17^GFP^ mice at 7, 10 and 30 dpi. Arrows indicate GFP^+ve^ cells. Scale bars = 100μm (left panels) and 15μm (right panels). Nuclei were labelled with Hoechst 33258 and muscle fibers labelled using Phalloidin. Images representative of 3 independent experiments (n = 5 mice per group).

To visualise IL-17–producing infiltrating cells in the feet and muscle of RRV-infected IL-17^GFP^ mice, we collected tissues at 7, 10 and 30 dpi and processed them for wholemount or thick-tissue sectioning and immunofluorescence microscopy. Confocal imaging of foot tissue showed large infiltrates of GFP^+^ cells at 10 dpi and collagen IV staining showed substantial remodelling of vasculature in the interarticular muscle of inflamed feet (Fig 4A and S1 Movie). IL-17^GFP+ve^ CD4^+^ and CD8^+^ T cells were found in the interarticular muscle at 7 and 10 dpi (Fig 4B), rather than in the synovial space surrounding the bones of the ankle joint. As observed in Fig 3E, a number of GFP^+ve^ cells that were neither CD4^+^ or CD8^+^ were detected in the feet (Fig 4B). We performed immunostaining for neutrophils–a key infiltrating innate immune cell in inflammation–in the feet of RRV-infected mice (10 dpi) and observed that Ly6G^+^ neutrophils accounted for some of the IL-17^GFP+ve^ cells found in the inflamed foot joint during acute RRVD (Fig 4C). In addition, we found that some MHC-II^+ve^ cells co-expressed IL-17^GFP^ reporter signal in the interarticular muscle (S3 Fig) at 10 dpi, pointing to the possibility that myeloid cells such as macrophages may be a source, although not a major one, of the cytokine during peak RRVD. In the quadriceps muscle, IL-17^GFP+ve^ CD8^+^ T cells (but fewer CD4^+^ T cells) were detected during the acute phase at 7 and 10 dpi, and small clusters in the myofiber interstitial space were observed after resolution of disease at 30 dpi (Fig 5).

### Anti-IL-17 antibody treatment reduces arthritic disease severity in RRV-infected mice

**Fig 6:**
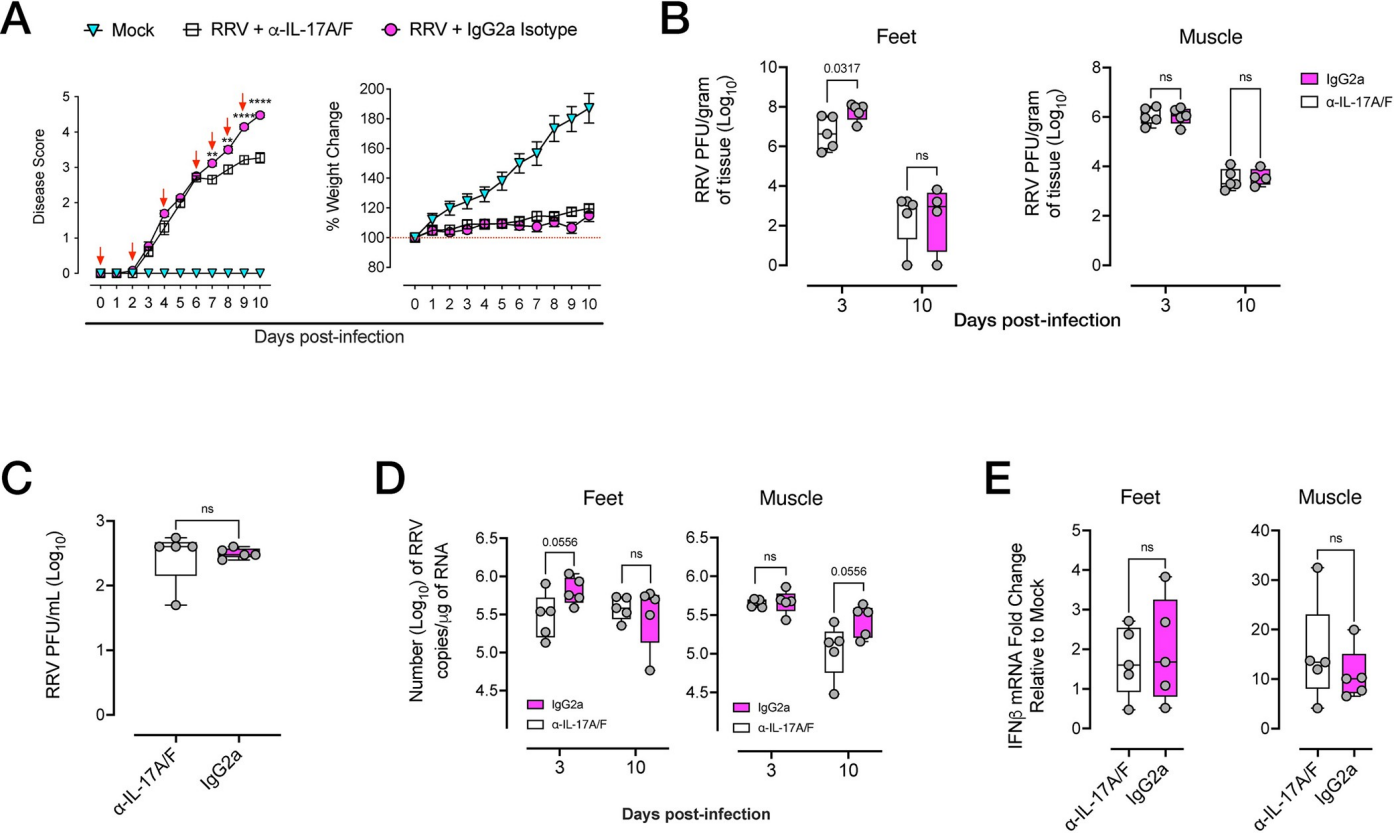
Modulation of IL-17 signaling ameliorates RRV disease. **A)** Disease and weight loss kinetics of RRV-infected C57BL/6J mice treated with anti-IL-17A/F monoclonal antibody (mAb; 2.86mg/kg) or with IgG2a isotype from 0, 2, 4 dpi and daily from 6 dpi to 9 dpi (red arrows show daily treatment). Data shown as mean +/- SEM. Statistically significant differences between groups determined by multiple *t*-test with Holm-Sidak correction. **, *p* < 0.01; ****, *p* < 0.0001. **B)** RRV T_48_ viral titers in the feet and quadriceps muscle of RRV-infected C57BL/6J mice treated with anti-IL-17A/F mAb or with IgG2a isotype, at 3 and 10 dpi. Tissue viral titers were normalised to tissue weight and expressed as PFU/per gram of tissue (PFU/g). **C)** RRV T_48_ viral titers in the serum of RRV-infected C57BL/6J mice treated with anti-IL-17A/F mAb or with IgG2a isotype at 3 dpi. **D**) RRV RNA load in the feet and quadriceps muscle of RRV-infected C57BL/6J mice treated with IL-17A/F mAb or with IgG2a isotype, at 3 and 10 dpi. Data represented as RRV RNA copy number (log_10_/μg of RNA). **E)** Fold change in *IFNβ* mRNA expression in the feet and muscle of RRV-infected C57BL/6J mice treated with IL-17A/F mAb or with IgG2a isotype, at 3 dpi. Data shown as fold change in expression compared to mock-infected mice, normalized to housekeeping gene expression. Data shown (mean +/- SEM) is representative of 3 independent experiments (n = 6 mice per group). Statistically significant differences between groups determined by Mann-Whitney U Test, *p* values indicated on graphs. Statistically significant differences shown as numerical p values (p<0.05). ns: not significant.

In light of our observations that IL-17–expressing immune cells were infiltrating the foot tissue during acute RRVD, we asked whether *in vivo* blockade of IL-17 signaling could reduce the severity of arthritic inflammation. Mice were administered anti-IL-17A/F monoclonal antibody (mAb) or IgG2a isotype control intraperitoneally (i.p.) at 0, 2, 4 dpi and daily from 6 dpi until 9 dpi at a dose of 2.86mg/kg. Mice were monitored for clinical signs of arthritic disease (using a scoring matrix described in Materials and Methods) and feet and quadriceps muscle were collected at 10 dpi. Mice treated with anti-IL-17A/F mAb showed a statistically significant reduction in disease severity compared to untreated mice, with significant reduction in clinical scores from 7 dpi to 10 dpi (Fig 6A). We next examined the effect of anti-IL-17A/F mAb on viral titers and replication in feet and quadriceps muscle. However, a significant reduction in titres was found in the feet, but not the muscle tissue, of mice treated with anti-IL-17A/F at 3 dpi. By 10 dpi, viral titres in feet and muscle were similar between anti-IL-17A/F–treated and untreated mice (Fig 6B). At 3 dpi, we found no differences in viral plaques in the serum of mice treated with anti-IL-17A/F compared to untreated mice (Fig 6C**).** Likewise, no differences in viral RNA load were found in the feet or muscle of mice treated with anti-IL-17A/F compared to that of isotype-treated mice at 3 dpi and 10 dpi, although a modest reduction was observed in the feet and muscle of mAb-treated mice at 3 dpi and 10 dpi, respectively (Fig 6D). In light of these data, and previous reports showing that in CHIKV infection, mice deficient in IL-17 signaling displayed an increase in Type I IFN responses that was associated with reduced viral burden [64], we asked whether anti-IL-17A/F treatment was also associated with enhanced, early Type I IFN transcriptional responses in the tissues. At 3 dpi, we observed no significant differences in IFNβ mRNA expression in the feet and muscle (Fig 6E). We also examined tissue mRNA expression of IRF7, a transcription factor essential for Type I IFN-dependent antiviral responses in alphavirus-induced arthritis, and found no statistically significant differences in induction at 3 dpi (S4A Fig). We also asked whether late Type I IFN stimulated genes (ISGs), of which the expression can persist beyond the early phase of antiviral responses, were impacted by treatment with anti-IL-17A/F mAb. We examined ISG15 mRNA expression at 3 dpi and 10 dpi in the feet and muscle, and found no statistically significant differences between anti-IL-17A/F–treated and untreated mice (S4B Fig).

**Fig 7:**
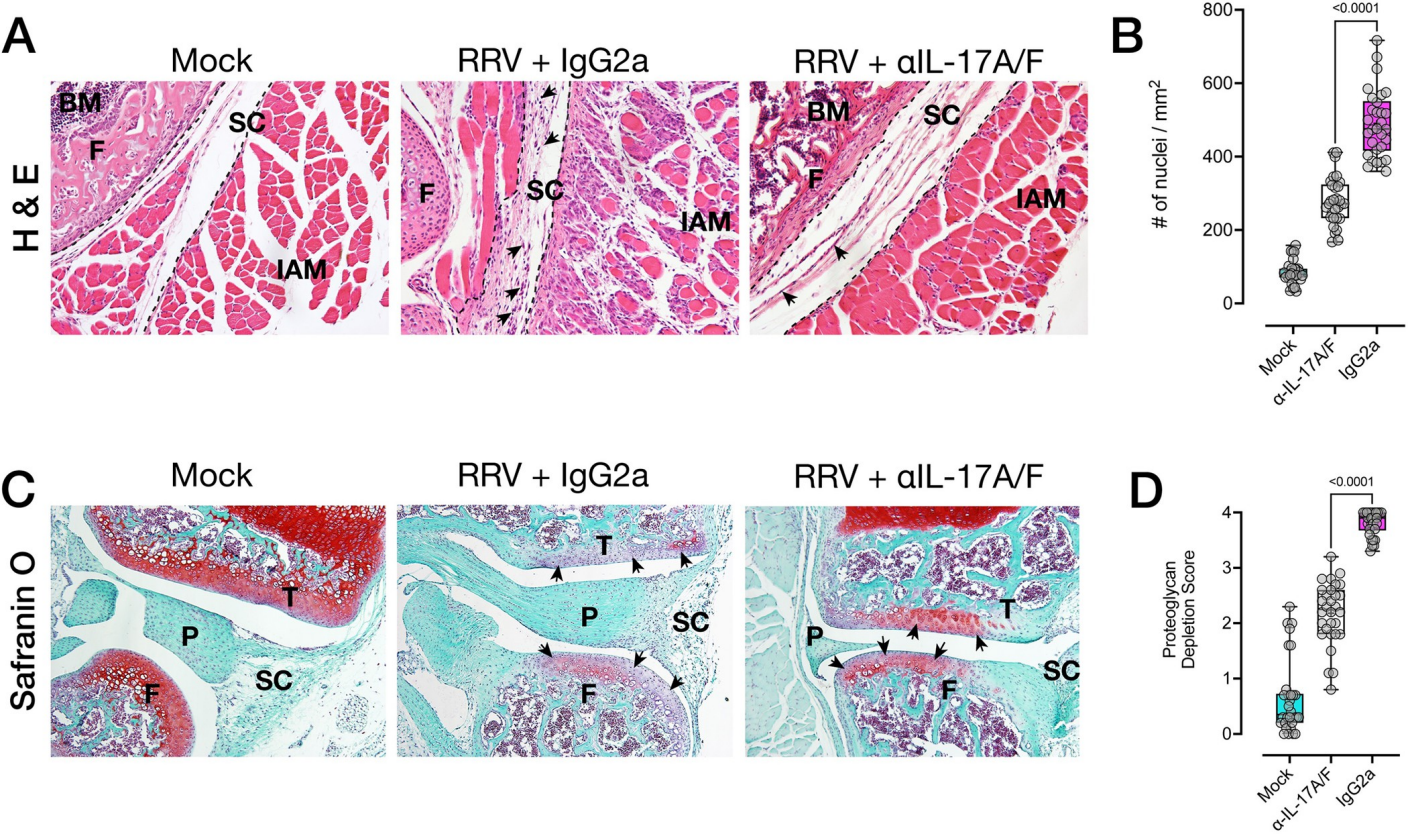
Anti-IL-17A/F monoclonal antibody reduces musculoskeletal disease in RRV infection. **A)** Representative hematoxylin and eosin (H&E) staining of paraffin sections of joint synovial space of mock-infected, RRV-infected C57BL/6J mice treated with IgG2a isotype and RRV-infected C57BL/6J mice treated with anti-IL-17A/F mAb, at 10 dpi. Arrows indicate infiltrating leukocyte aggregates in the synovium of the knee joint. **B)** Quantification of cellular infiltrates (automated nuclei count) of H&E–stained synovium as described in **A)**. Data are presented as the mean +/- SEM of 30 regions of interest (ROIs) per group. **C)** Representative safranin O staining of the femoral/tibial interface of the knee, at 10 dpi. Arrows indicate intensity of safranin O stain in the medial and lateral condyle of the femur and tibia. F: femur; T: tibia; BM: bone marrow; SC: synovial capsule; IAM: interarticular muscle; P: pannus. **D)** Proteoglycan depletion score of safranin O–stained knee joint sections from RRV-infected C57BL/6J mice treated with anti-IL-17A/F mAb, at 10 dpi, as described in **C)**. Data presented as the median +/- SEM of 30 ROIs per group from 2 pooled experiments (NE). Data shown as box-whiskers plots (bar shows median, + shows mean, error bars show range; n = 5 mice per group, NE = 2). Statistically significant differences between groups were determined with Mann-Whitney U test, *p* values indicated on graphs. Images acquired with a 20X objective.

**Fig 8:**
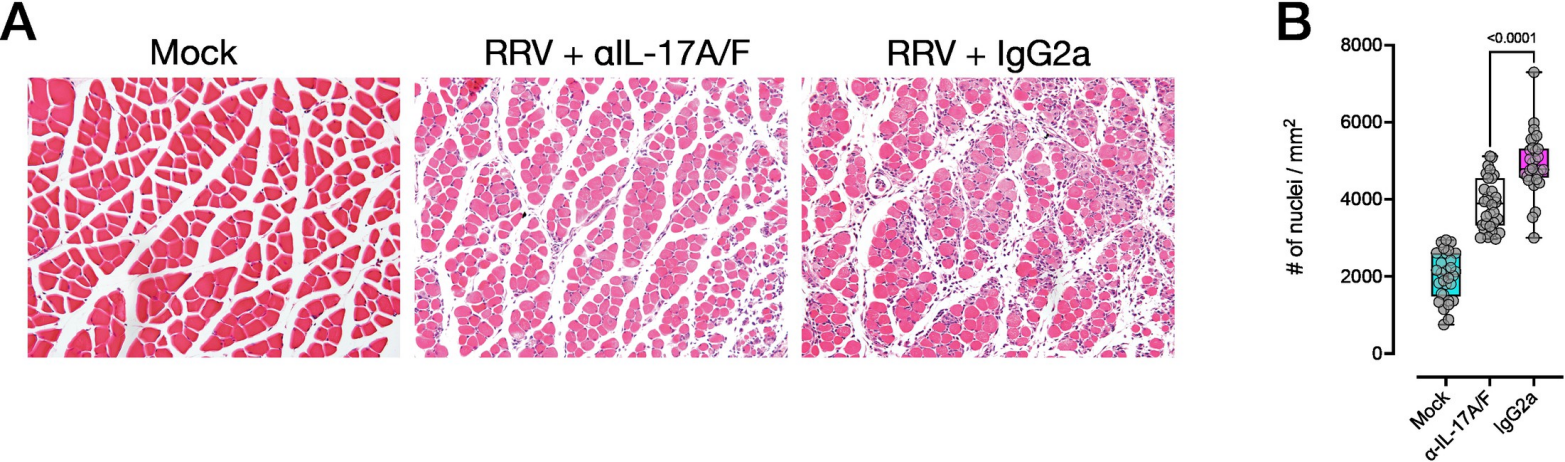
Anti-IL-17A/F monoclonal antibody reduces muscle inflammation in RRV infection. **A)** Hematoxylin and eosin (H&E) staining of paraffin sections of quadriceps muscle tissue of mock-infected, RRV-infected mice and RRV-infected C57BL/6J mice treated with anti-IL-17A/F mAb, at 10 dpi (20X objective). **B)** Quantification of cellular infiltrates (automated nuclei count) of H&E–stained muscle, as described in **A)**. Data are presented as the mean +/- SEM of 30 regions of interest (ROIs) per group. All data representative of n = 5 mice per group, NE = 2. Statistically significant differences between groups were determined with Mann-Whitney U test, *p* values indicated on graphs.

We next assessed histopathology of inflamed tissues during peak RRVD (10 dpi). Haematoxylin and eosin (H&E) staining of the joints showed a reduction in overall infiltrating leukocytes in mice treated with anti-IL-17A/F mAb, including in the interarticular muscle and synovium of the knee joint (Fig 7A). Automated quantification of cellular infiltrates across multiple, non-consecutive tissue sections confirmed a significant reduction in tissue infiltration in the knee joint (Fig 7B). In addition, we used safranin O staining to assess cartilage damage in RRV-infected mice treated with anti-IL-17A/F mAb and those given IgG2a isotype. Safranin O staining intensity at the femoral/tibial interface showed substantial cartilage matrix erosion in mice given IgG2a isotype (Fig 7C), whereas clear staining of cartilage proteoglycan was readily visible in the anti-IL-17A/F–treated group. We evaluated differences in safranin O staining using a semi-quantitative proteoglycan depletion score [65], and found a statistically significant reduction in cartilage erosion in mice treated with anti-IL-17A/F mAb (Fig 7D). Altogether, this data suggests that the proinflammatory environment in inflamed tissues, as well as overall musculoskeletal and synovial pathology–including cartilage damage–is dampened in RRV-infected mice treated with anti-IL-17A/F mAb. We found similar reductions in cellular infiltrates in the quadriceps muscle of mice treated with anti-IL-17A/F mAb (Fig 8A and 8B) compared to mice given IgG2a.

### Anti-IL-17 antibody treatment modulates cellular and soluble host factors in RRV-infected mice

**Fig 9:**
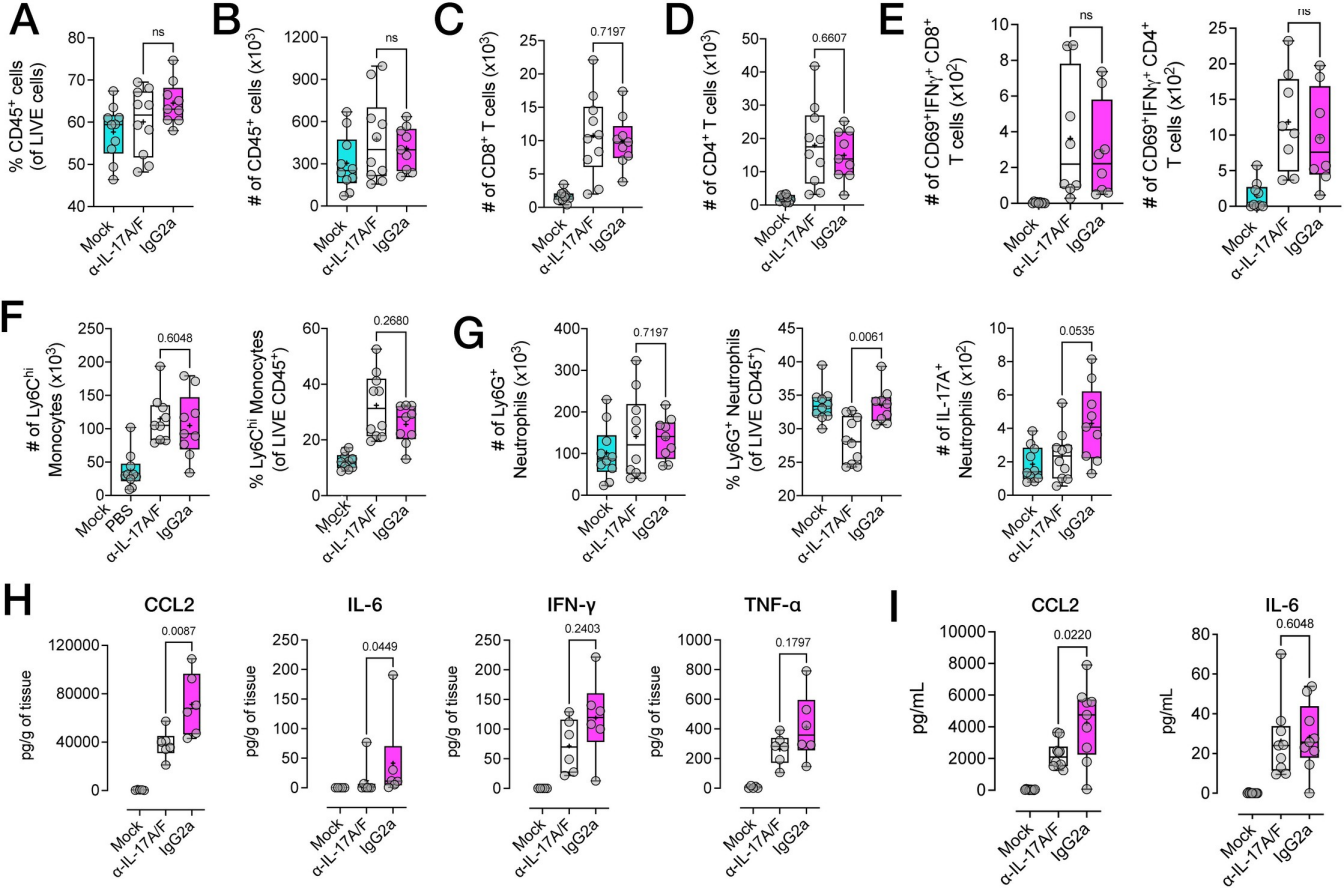
Modulation of IL-17 in RRV disease disrupts myeloid, but not lymphoid cell infiltration into the joint tissue. **A**) Proportions and **B**) numbers (of total LIVE cells) for infiltrating CD45^+^, **C**) CD8^+^ and **D)** CD4^+^ T cells (gated from LIVE CD45^+^Ly6G^-^CD11b^-^CD3^+^ cells) in the feet of RRV-infected C57BL/6J mice treated with anti-IL-17A/F mAb at 10 dpi. **E**) Numbers of activated CD69^+^IFNγ^+^ CD8^+^ and CD4^+^ T cells in the feet of RRV-infected C57BL/6J mice treated with anti-IL-17A/F mAb at 10 dpi. **F)** Total number and proportions (% of LIVE CD45^+^ cells) of inflammatory Ly6C^hi^ monocytes and **G)** total numbers and proportions (% of LIVE CD45^+^ cells) of Ly6G^+^ neutrophils in the feet of RRV-infected mice treated IL-17A/F mAb at 10 dpi. Data presented as numbers (per paired feet) of LIVE CD45^+^Ly6G^-^CD11b^+^Ly6C^hi^ for monocytes, and LIVE CD45^+^Ly6G^+^ for neutrophils, respectively **H**) Number of IL-17A^+^ neutrophils in the feet of RRV-infected mice treated IL-17A/F mAb at 10 dpi. Data presented as absolute cell numbers of LIVE CD45^+^CD3^-^Ly6G^+^IL-17A^+^ cells. Data in **A**-**G** shown as box-whiskers plots (bar shows median, + shows mean, error bars show range), presented as mean +/- SEM (n = 5 mice per group, NE = 2). Statistically significant differences between groups determined with Mann-Whitney U test, *p* values indicated on plots. **I)** Soluble cytokine protein levels in the feet of RRV-infected C57BL/6J mice treated with anti-IL-17A/F mAb or IgG2a isotype, at 10 dpi. Data normalised to tissue weight and shown as pg/gram of tissue (pg/g). **J)** Serum levels of CCL2 and IL-6 of RRV-infected C57BL/6J mice treated with anti-IL-17A/F mAb or IgG2a isotype, at 10 dpi. Data presented as pg/mL. Data shown as box-whiskers plots (bar shows median, + shows mean, error bars show range), presented as mean +/- SEM, (n = 5 mice per group, NE = 2). Statistically significant differences between groups determined with Mann-Whitney U test, *p* values indicated on graphs.

To understand how anti-IL-17A/F mAb treatment may have contributed to reduced disease severity, we examined the immune cell composition of inflamed feet using flow cytometry. At 10 dpi, there were no significant differences in the number and proportions of CD45^+^ infiltrating cells, (Fig 9A and 9B), and the number of infiltrating CD8^+^ or CD4^+^ T cells (Fig 9C and 9D) was similar between anti-IL-17A/F–treated and isotype-treated mice. There were also no differences in the number of infiltrating activated CD69^+^IFNγ^+^ T cells in the feet (Fig 9E). Total numbers of infiltrating inflammatory CD11b^+^Ly6C^hi^ monocytes and Ly6G^+^ neutrophils were likewise unaffected (Fig 9F and 9G). However, when assessing the relative composition of infiltrating CD45^+^ leukocytes, we found that the proportion of Ly6G^+^ neutrophils (of all CD45^+^ leukocytes) was significantly reduced in the feet of mice treated with anti-IL-17A/F mAb (Fig 9E) and interestingly, the number of IL-17A^+^ neutrophils was also reduced in this tissue (Fig 9H). Of note, the proportion of inflammatory Ly6C^hi^ monocytes was moderately increased in mice treated with anti-IL-17A/F mAb compared to those that received IgG2a isotype (Fig 9F). We further examined the phenotype of these myeloid infiltrating cells (S5A Fig) found that within these Ly6C^hi^ monocytes, the proportion of CCR2^+^CX_3_CR1^+^ monocytes was significantly elevated in mice treated with anti-IL-17A/F (S5A and S5B Fig), and the proportion of CX_3_CR1^+^ CD64^+^ synovial macrophages (S 5A and S5C Fig) was significantly higher in mice treated with anti-IL-17A/F.

**Fig 10:**
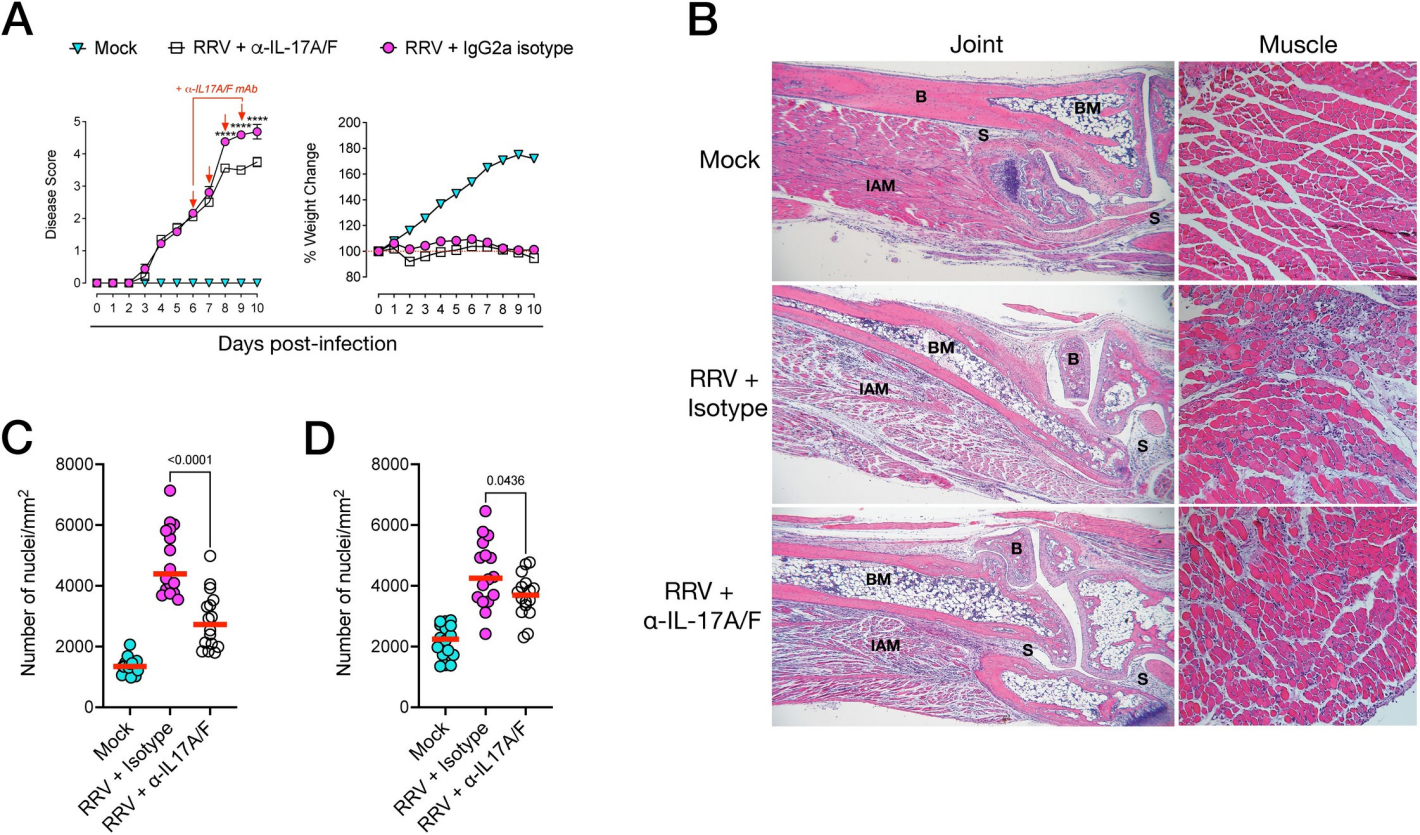
Therapeutic anti-IL-17A/F monoclonal antibody treatment reduces musculoskeletal disease in RRV infection. A) Disease score and weight loss kinetics of RRV-infected C57BL/6J mice treated with anti-IL-17A/F monoclonal antibody (mAb) or with IgG2a isotype from 6, 7, 8 and 9 dpi (red arrows show daily treatment). Data presented as mean +/- SEM. Statistically significant differences between groups determined by multiple *t*-test with Holm-Sidak correction. ****, *p* < 0.0001. **B)** Hematoxylin and eosin (H&E) staining of paraffin sections of joint (left) and quadriceps muscle (right). B: bone; BM: bone marrow; S: synovium; IAM: interarticular muscle (left; 4X objective, right; 20X objective). **C)** Quantification of cellular infiltrates (automated nuclei count) of H&E–stained joint (interarticular muscle) and **D)** quadriceps muscle, as described in **B)**. Data are presented as the mean +/- SEM of 16 regions of interest (ROIs) per group. Data shows 2 independent experiments (n = 5 mice per group). Statistically significant differences between groups were determined with Mann-Whitney U test, *p* values indicated on graphs.

Next, we quantified levels of soluble proinflammatory cytokines and chemokines in the feet. At 10 dpi, CCL2 and IL-6 protein levels were significantly reduced in the feet of anti-IL-17A/F-treated mice, whereas IFNγ and TNF levels were modestly reduced (Fig 9I). In the serum, CCL2 levels were significantly reduced in mice treated with anti-IL-17A/F mAb, while IL-6 levels remained unchanged (Fig 9J). Of note, when anti-IL-17A/F mAb was administered therapeutically, from 5 dpi to 9 dpi at a daily dose of 3.5mg/kg, a reduction in disease was also observed (Fig 10A), and was accompanied by a significant reduction in leukocyte infiltrates in both joint interarticular muscle (IAM) and quadriceps muscle tissue (Fig 10B). Automated quantification of cellular infiltrates confirmed a significant reduction in infiltration in the foot joint (Fig 10C) and muscle tissues (Fig 10D).

### Anti-IL-17A/F antibody treatment modulates immunometabolic pathways in the musculoskeletal microenvironment during acute RRV disease

**Fig 11:**
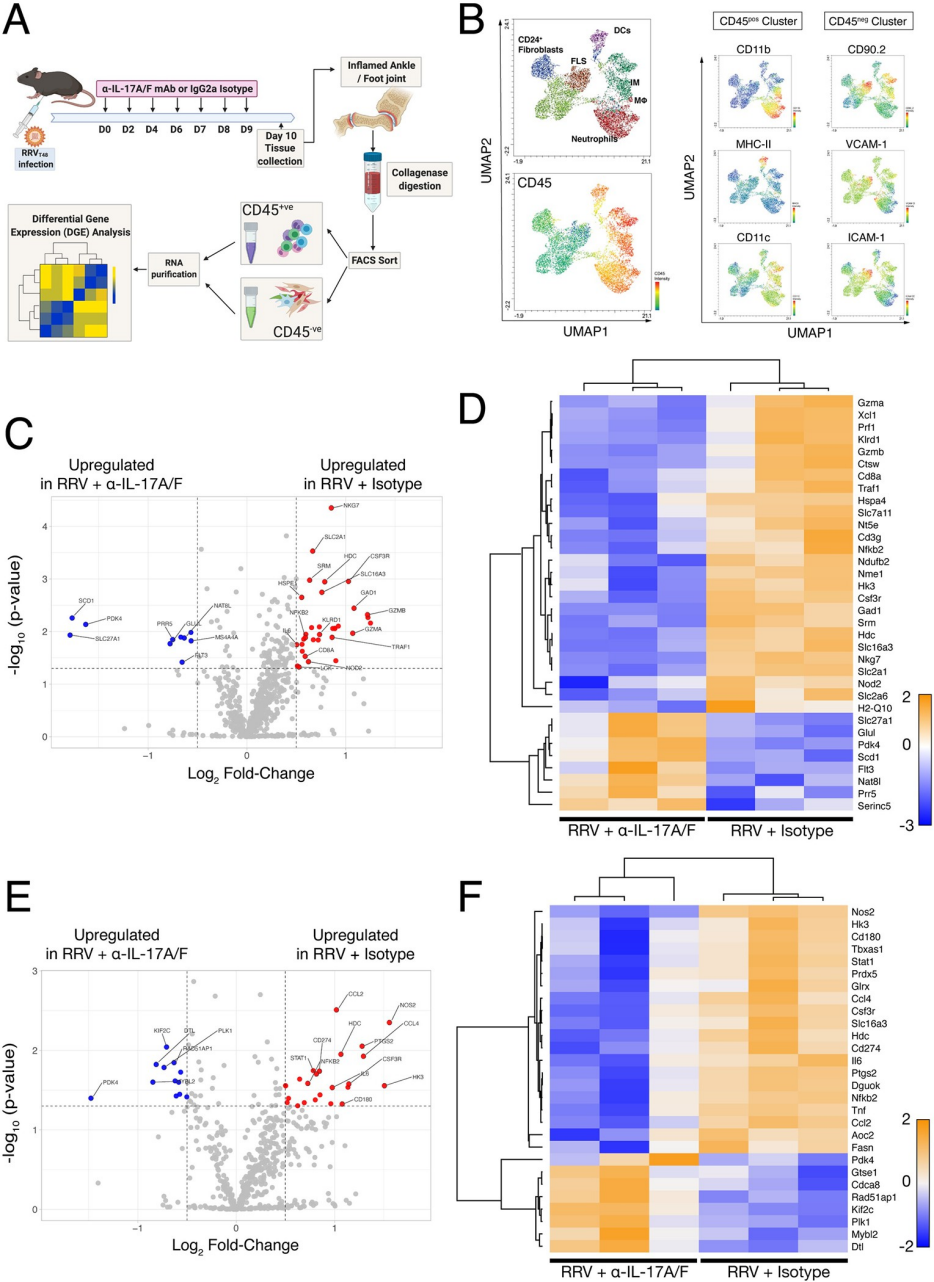
Treatment with anti-IL-17A/F monoclonal antibody shifts transcriptional profile of the musculoskeletal microenvironment in RRV infection. **A)** Schematic of isolation of CD45^+^ and CD45^-^ musculoskeletal cells from RRV-infected C57BL/6J mice treated with anti-IL-17A/F or IgG2a isotype control antibodies at day 10 post-infection prior to cell sorting and Nanostring analysis. Gating strategy for CD45^+^ and CD45^-^ cell sorting shown in S6 Fig. **B)** Flow cytometry analysis of musculoskeletal CD45^+^ and CD45^-^ compartments from RRV-infected C57BL/6J mice at 10 dpi visualized with UMAP dimensional reduction. CD45 intensity shown as per intensity heatmap, and expression intensity of CD11b, MHC-II and CD11c, and CD90.2, VCAM-1 and ICAM-1 shown for CD45^+^ and CD45^-^ clusters, respectively. **C)** Volcano plot of differentially expressed genes in the CD45^+^ compartment isolated from the feet of RRV-infected mice C57BL/6J treated with anti-IL-17A/F or isotype control at 10 dpi (blue dots = genes upregulated in anti-IL-17A/F-treated mice compared to isotype-treated mice; red dots = genes upregulated in isotype-treated mice compared to anti-IL-17A/F-treated mice). **D)** Heatmap of unsupervised hierarchical clustering of differentially regulated genes in the CD45^+^ compartment in the feet of RRV-infected C57BL/6J mice treated with anti-IL-17A/F or isotype control, at 10 dpi. **E)** Volcano plot of differentially expressed genes in the CD45^-^ compartment isolated from the feet of RRV-infected mice C57BL/6J treated with anti-IL-17A/F or isotype control at 10 dpi (blue dots = genes upregulated in anti-IL-17A/F-treated mice compared to isotype-treated mice; red dots = genes upregulated in isotype-treated mice compared to anti-IL-17A/F-treated mice). **F)** Heatmap of unsupervised hierarchical clustering of differentially regulated genes in the CD45^-^ compartment in the feet of RRV-infected C57BL/6J mice treated with anti-IL-17A/F or isotype control, at 10 dpi. Data are representative of 1 independent experiment (n = 3 mice per group).

In light of our observations that anti-IL-17A/F mAb treatment results in the amelioration of arthritic disease in RRV-infected mice, we asked how disrupting the IL-17 pathway impacted the transcriptional profile of joint cells within both the cellular infiltrates and the musculoskeletal stromal compartment. Mice were infected with RRV and treated with either anti-IL-17A/F or IgG2a isotype, and at 10 dpi, single-cell suspensions were isolated from the inflamed feet and FACS-sorted (gating strategy shown in S6 Fig) into CD45^+^ (leukocyte) and CD45^-^ (stromal) fractions (Fig 11A). mRNA was extracted from these samples and differential gene expression (DGE) were assessed via Nanostring analysis using an immune response and immunometabolism gene panel. We asked whether the CD45^+^ and CD45^-^ cell populations clustered as two distinct compartments, and used UMAP reduction to identify stromal and myeloid cells in the feet of RRV-infected mice (Fig 11B). The CD45^+^ cluster included neutrophils, dendritic cells, monocytes and macrophages, and the CD45^-^ cluster included populations of CD90.2^+^ ICAM-1^+^ and VCAM-1^+^ fibroblast-like synoviocytes (FLS) and CD24^+^CD90.2^int^ synovial fibroblasts: this indicated that using CD45 as a marker to sort musculoskeletal cell suspensions from RRV-infected mice could shed some light on how both immune and non-immune cell compartments are a transcriptionally regulated following anti-IL-17A/F treatment.

In the CD45^+^ compartment, there was a substantial enrichment of proinflammatory gene signatures in the IgG2a isotype-treated, RRV-infected samples (*red dots*) compared to CD45^+^ cells in IL-17A/F–treated mice (*blue dots*; Fig 11C). In particular, genes associated with innate immune signaling, such as CSF3R (G-CSF receptor), interleukin-6 (IL6) and TRAF1 (TNF receptor-associated factor 1), and genes associated with T cell and natural killer (NK) cytolytic function, such as PRF1 (perforin), GZMA, GZMB (Granzyme A and B, respectively) and KLRD1 were upregulated. Genes that regulate effector T cell function such as CD8A and LCK were likewise highly enriched in infiltrating leukocytes from IgG2a isotype-treated mice (Fig 11C). In contrast, CD45^+^ cells from IL-17A/F–treated mice (*blue dots*) showed enrichment of metabolic gene signatures associated with homeostasis and regulation of inflammation (Fig 11C), and unsupervised hierarchical clustering of the gene signatures in the CD45^+^ compartment revealed a distinct downregulation of proinflammatory gene signatures in the feet of IL-17A/F-treated mice (Fig 11D).

Interestingly, DGE analysis of the CD45^-^ (*ie*., stromal) compartment likewise showed a clear shift away from proinflammatory gene signatures in mice treated with IL-17A/F mAb, with a clear downregulation in expression of proinflammatory genes such as CCL2, IL-6, CCL4 and and NOS2 (Fig 11E), which are known effector cytokines in alphavirus-induced inflammation [66–72]. As was observed with cells from the CD45^+^ compartment using unsupervised hierarchical clustering of gene expression, IL-17A/F treatment exerted a pronounced shift in the transcriptional profile of musculoskeletal stromal cells, in particular with genes associated with inflammatory responses (Fig 11F). In both CD45^+^ and CD45^-^ compartments, IL-17A/F treatments led to an enrichment of a small number of genes associated with metabolic regulation of homeostasis and inflammation. In CD45^+^ cells, genes such as SLC27A1 (or FATP1) and NAT8L were upregulated following anti-IL-17A/F treatment. PRR5, a component of the mTOR pathway involved in promotion of bone formation and skeletal growth, was also upregulated following anti-IL-17A/F treatment. In the CD45^-^ compartment, genes required for cell survival, proliferation and DNA strand repair such as MYBL2, PLK1, PDK4 and RAD51AP1 were upregulated following anti-IL-17A/F treatment. This data provides further information on the mechanisms underlying the therapeutic effect of anti-IL-17A/F mAb treatment in RRV-infected mice, and supports the hypothesis that interfering with a cytokine signaling pathway leads to a broader host response in both leukocyte and stromal cell compartments.

## Discussion

In this study we show that IL-17, a cytokine that has been shown to be important in arthritic pathologies such as rheumatoid arthritis (RA) and psoriatic arthritis (PsA) [44,52,73], plays a role in alphavirus-induced musculoskeletal pathology. Alphavirus-induced arthritis shares several pathophysiological features with RA, from synovitis to pain and cartilage erosion [56]. IL-17 is a key contributor to arthritic inflammation in RA and PsA [44,52,73], and can exacerbate tissue inflammation by upregulating proinflammatory cytokines such as TNF and IL-1β, and contribute to osteoclastogenesis and bone resorption by promoting the expression of RANKL [74,75].

In humans, Ross River virus disease (RRVD) is generally characterised by severe arthritis, primarily affecting the joints, hands and knees [1]. However, most experimental studies that use the disease model of RRV infection focus on inflammatory processes that affect the muscle tissue, likely due to technical limitations inherent to the extraction of cells from the joint synovium. Focusing our investigation on foot joint synovium, we showed that both CD4^+^ and CD8^+^ T cells infiltrated the interarticular muscle space of the foot during the acute phase, and persisted after resolution of inflammation and viral clearance. Although CD8^+^ T cells have been shown to be important–but not essential–in clearance of RRV [14], this is consistent with findings in CHIKV infection models that T cells also play a role in pathogenesis [15,18,19]. In this study, our findings that IL-17 expression was also observed in patients suffering from acute RRVD is corroborated by the increased levels of *Il17a* gene expression in both feet and muscle tissue during acute RRVD in our mouse model. Interestingly, T cells–including IL-17–producing T cells–were also found to persist in the interarticular space of the feet in RRV-infected mice up to 30 dpi, well beyond resolution of inflammation, although these cells did–at this time point–express classical tissue-resident memory (T_RM_) T cell phenotype. Whether this may be due to the timing post-infection, the quality of the antigenic signal or the nature of tissue-specific phenotypic markers of T_RM_ are important questions that deserve investigation. Our findings could be of relevance to the question of pathogenic T cell persistence in synovial tissue, which has been associated with chronic arthritic disease such as RA [76].

Interestingly, CD4^+^ and CD8^+^ T cells in the feet during the acute phase of disease expressed both IL-17A and IL-17F, indicating that IL-17RA signaling may require binding of both IL-17A and IL-17F isoforms in the context of alphavirus-induced arthropathy. While numbers of IL-17–producing γδ T cells in the feet were negligible, we found that neutrophils as well as MHC-II^+ve^ myeloid cells also expressed IL-17 in the feet during peak RRV disease, corroborating previous reports that neutrophils can contribute IL-17 in RA [77], and pointing to the possibility that synovial macrophages could be contributing to this signal in an highly inflamed environment, as has been shown in other disease models [78,79].

In light of data indicating that RRVD and IL-17 expression in tissues were concordant, we asked whether blocking IL-17 signalling using a monoclonal anti-IL-17A/F antibody would alter the course of RRVD. RRV-infected mice treated with anti-IL-17A/F monoclonal antibody (mAb) developed less severe arthritic disease and consistently showed reduced clinical disease scores compared to RRV-infected mice that received IgG2a isotype control. This was observed from the onset of acute RRV disease (7 dpi), which is consistent with previous reports using immunomodulatory approaches in RRVD, where significant reduction in disease severity is observed from the onset of disease [11,80]. Importantly, IL-17A/F–treated mice did not develop higher viral titers or viral RNA load in muscle and foot tissues, whether in the early (3 dpi) or acute (10 dpi) phase of RRVD. Interestingly, treatment with IL-17A/F mAb did not affect early (3 dpi) mRNA expression of IFNβ or IRF7, nor that of ISG15 both at 3 and 10 dpi. This is of interest as a study reported that abrogation of IL-17A signalling restored robust antiviral responses in CHIKV infection, where IL-17A was found to inhibit the expression of Type I IFN in an IRF5/IRF7-dependent manner [64].

Our findings differ from this study in that therapeutic treatment with anti-IL-17A/F did not positively regulate Type I IFN signalling, and was only associated with a modest reduction in viral titres in the feet at 3 dpi, but not 10 dpi. Several factors could explain these differences, including 1) the use of *in vitro* cell cultures—as opposed to *in vivo* disease model—to determine the impact of IL-17A signalling blockade on Type I IFN induction at a cellular level, 2) the possibility that CHIKV—although an Alphavirus related to RRV—may stimulate more robust IL-17A induction which, in turn, may effect downregulation of Type I IFN signalling, and 3) in constitutive IL-17A^-/-^ and IL-17RA^-/-^ knock-out mice, any positive feedback loop linking IL-17A signalling and Type I IFN induction may be completely abrogated, whereas treatment with anti-IL-17A/F mAb may only partially disrupt this system. Nevertheless, our findings have key implications in the clinical context, as interfering with immune signalling pathways *in vivo* may compromise host antiviral responses: here, we find that anti-IL-17A/F treatment during the development of RRV disease does not compromise Type I IFN signalling, nor does it provide an advantage to viral replication and dissemination.

Analysis of the cellular infiltrate composition in the feet of RRV-infected mice revealed that *in vivo* blockade of IL-17A/F did not have a significant impact on overall numbers of CD4^+^ and CD8^+^ T cells, and was not associated with a reduction in overall numbers of inflammatory Ly6C^hi^ monocytes and Ly6G^+^ neutrophils. However, the proportion of Ly6G^+^ neutrophils (of CD45^+^ leukocytes), and IL-17A^+^ neutrophils in particular, found in the feet was significantly reduced in mice treated with anti-IL-17A/F. Though we initially hypothesised that T cells were the primary source of IL-17 in RRV pathogenesis, our data shows that anti-IL-17 treatment may have a more profound impact on neutrophils in the context of arthritic inflammation. The association between neutrophils and IL-17 has been the subject of recent studies that showed that neutrophils can, in RA, be the main producers of IL-17 [77]. In addition, recent work on CHIKV arthritis suggests a strong association between neutrophil-driven tissue inflammation and IL-17 signalling [81,82]. This is particularly relevant as one of the observed effects of anti-IL-17A/F treatment was the reduction of CCL2 and IL-6 protein expression in the feet and in the serum, as well as IFNγ and TNF, albeit to a lesser extent. The role of IL-6 in the context of pathogenic neutrophils should not be disregarded, as studies showed that in skin inflammation, IL-17A^+^ neutrophils depend on IL-6 signalling [83], which may account for earlier findings that *in vivo* modulation of IL-6 signalling using a mAb reduces RRV disease in mice [84].

An interesting observation was the increased proportion of CX_3_CR1^+^CCR2^+^Ly6C^hi^ monocytes in the feet of mice treated with anti-IL-17A/F. A study showed that CCR2^+^Ly6C^hi^ inflammatory monocytes are essential for antiviral responses and viral clearance in RRV infection [85] despite their role in inflammation and pathology, and conditional depletion of these cells was detrimental to the mice. Myeloid cells such as monocytes and macrophages can respond to their microenvironment by adopting tissue repair-promoting phenotypes, of which expression of surface markers like CX_3_CR1 is a feature. Previous work showed that in RRV-infected mice, local accumulation of CX_3_CR1^+^CD64^+^ macrophages can help promote muscle tissue repair in RRV-infected mice [86]. Here, we observed the establishment of CX_3_CR1^+^CD64^+^ macrophages in the feet of mice treated with anti-IL-17A/F mAb, which could be associated with reduced pathology and tissue damage. There is little information on the relationship between IL-17 signaling and synovial macrophages in viral arthritis, but our findings suggest that anti-IL-17A/F treatment, and the resulting decrease in CCL2 and IL-6 during acute RRV disease may facilitate the establishment of CX_3_CR1^+^ monocytes and macrophages in the synovium.

While a reduction in proinflammatory protein levels or mRNA expression in tissues may point towards a causative effect following treatment, these only provide partial mechanistic information. Massive leukocyte influx, myeloid cell-induced tissue damage and proinflammatory cytokine production by infiltrating leukocytes are key factors in RRV-induced pathology, yet much less is known on how the synovial stroma responds following infection and inflammation. Following our observations that anti-IL-17A/F mAb treatment reduces synovial inflammation and ameliorates RRVD, we asked how anti-IL-17 treatment impacted the transcriptional signature of musculoskeletal stromal cells, which encompass fibroblasts, synoviocytes, mesenchymal stem cells as well as tissue-resident macrophages. By analysing the immune and metabolic transcriptional profiles of both inflammatory (CD45^+^) and stromal (CD45^-^) compartments, we observed that treatment of RRV-infected mice with anti-IL-17A/F mAb drove profound changes in the gene signature of the inflamed musculoskeletal microenvironment.

We showed that anti-IL-17 treatment led to a profound shift away from proinflammatory gene signatures in infiltrating CD45^+^ leukocytes, as shown by the downregulation of genes that govern both innate and adaptive effector functions. In particular, GZMA and GZMB which encode for Granzymes A and B, respectively, have been shown to drive acute CHIKV arthritis both in mice and humans [69,87]. In our study, treatment with anti-IL-17A/F mAb also led to a significant downregulation of genes required for NK cell (NKG7, KLRD1) [88] and T cell (CD8A, LCK) [89] effector function, as well as the gene encoding perforin (PRF1), a key component of cytolytic function in NK cells and T cells [90].

The distinct shift from a proinflammatory gene signature following IL-17A/F treatment was also observed in the CD45^-^ musculoskeletal compartment. Of particular relevance to the established link between IL-6 signaling and IL-17–producing neutrophils, expression of CCL2, and IL-6 was significantly downregulated in the CD45^-^ fraction, corroborating our data showing that CCL2 and IL-6 protein levels were reduced in the serum. In addition, gene expression of NOS2, CCL4 (a CCR5 ligand) and CSF3R (G-CSF receptor) were downregulated in IL-17A/F-treated mice. These genes encode for potent proinflammatory cytokines that are implicated in alphaviral arthritis, and their downregulation has been observed in studies where immunomodulatory intervention led to improved disease phenotypes [82,84,86,91].Together with the downregulation of transcription factors that control cellular antiviral responses such as NFKB2 and STAT1, which are needed for Type I interferon responses and subsequent proinflammatory processes and are essential in IL-17A signaling [92], our data supports the hypothesis that disrupting the IL-17 signaling pathway results in broad transcriptional changes that help dampen local synovial inflammation. Our transcriptional analysis also showed that in reducing local synovial inflammation, treatment with IL-17A/F mAb was associated with the upregulation of genes associated with metabolic regulation of immune responses, and this was observed in both CD45^+^ and CD45^-^ compartments.

RRV infection has been shown to promote glycolytic pathways *in vitro*, however a clear relationship between glycolytic pathways and IL-17 signaling in arthritic disease remains to be established [93]. In addition, studies have shown that differentiation and proliferation of IL-17–producing Th17 cells from naïve CD4^+^ T cells requires significant cellular metabolic changes, and these changes are essential in nutrient-poor environments such as the acidic environment found in inflamed RA joints [94,95]. Among genes upregulated in CD45^+^ cells from IL-17A/F mAb-treated mice, SLC27A1 encodes FATP1 (fatty acid transport protein-1), which helps control lipid and glucose metabolism, and has been associated with anti-inflammatory phenotypes in macrophages by acting as a modulator of oxidative stress [96], and is highly expressed in skeletal tissues during steady-state [97,98]. Likewise, SCD1 which was upregulated in leukocytes from IL-17A/F mAb-treated mice, encodes acyl-CoA desaturase-1, required for fatty acid metabolism and has been associated with reduced synovial inflammation in experimental models of RA [99].

Within the CD45^-^ population, MYBL2, a transcription factor expressed in proliferating cells [100], was upregulated in anti-IL-17A/F–treated mice. MYBL2 regulates cell cycle progression, cell differentiation and survival [101] by promoting cell cycle progression [102]. In addition, PLK1, a serine/threonine kinase that aids in cell-cycle progression, regulates mitotic processes, cytokinesis and cell proliferation [103,104], was upregulated in anti-IL-17A/F–treated mice. PLK1 is present in proliferative tissues such as the bone marrow, and its overexpression promotes cell proliferation [103,105] and was shown to partly rescue proliferation inhibition in an *in vitro* sepsis model [103].

In the CD45^-^ fraction isolated from untreated, RRV-infected mice, CD180, a TLR-like protein mainly expressed on antigen-presenting cells such as macrophages, myeloid dendritic cells and B cells [106], was upregulated. CD180 contributes to the homeostasis and survival of these cells, and promotes TLR4 signaling-mediated activation and proliferation of B cells, whilst inhibiting TLR4 signaling in macrophages and dendritic cells [107–109]. In the same group, SLC16A3, a lactate transporter expressed by macrophages and neutrophils, was also upregulated [110,111]. SLC16A3 is highly expressed by synovial fibroblasts (FLS) in RA synovial tissue, and silencing SLC16A3 inhibited the proliferation of RA-derived FLS and reduced disease severity in a RA mouse model [112]. Differential regulation of these genes suggests that anti-IL-17A/F treatment in RRVD may regulate transcriptional pathways associated with tissue homeostasis and cell proliferation. Further investigations on the role of IL-17A signaling in these tissue-specific transcriptional mechanisms may help unveil new therapeutic approaches.

Genes associated with cartilage homeostasis were also upregulated in infiltrating leukocytes isolated from IL-17A/F–treated mice: PDK4, which encodes pyruvate dehydrogenase kinase 4, is required for chondrocyte proliferation and is suppressed in RA joints [113], while GLUL, which encodes glutamine synthetase, has been shown to be downregulated in cartilage from patients with severe osteoarthritis [114]. This information is of particular relevance as our data also indicates that cartilage damage is substantially reduced in RRV-infected mice treated with anti-IL-17A/F antibody.

Our understanding of how immunometabolic regulation governs alphaviral disease is limited, but in RA, the synovial microenvironment is subjected to hypermetabolic changes due to high energy demand, and surges in infiltrating immune cells induces local production of cytokines and transcription factor-activating metabolites. This drives joint tissue-resident immune cells to acquire proinflammatory effector functions [115]. Some metabolic pathways, such as the glycolysis pathway, are important in maintaining this inflammatory state, as studies showed that metabolites of the glycolysis pathway were increased in the serum of RA patients [116], suggesting that changes in metabolism are associated with joint inflammation. A number of treatment interventions that modulate metabolism have been shown to have a therapeutic effect in RA (reviewed in [117]), a prime example of which is methotrexate (MTX). MTX has been used as a successful mono- or combination treatment to alleviate chronic symptoms in CHIKV patients [17,118–120] including a randomized controlled trial [121]. However, MTX was found to increase disease severity and onset in RRV-infected mice and increase viral load in quadriceps and serum [122]. Importantly, in the presence of anti-rheumatic drugs that target immunometabolic pathways (such as MTX), IL-17 signaling amplifies neutrophil chemotactic protein (IL-8, CXCL1 and CXCL2) expression in fibroblast-like synoviocytes, pointing towards a key role for musculoskeletal stroma in regulating inflammatory chemokine expression during joint inflammation. Therefore, the strong shift towards anti-glycolytic pathways in the CD45^-^ fraction we observed following treatment with anti-IL-17A/F, may have led to the downregulation of several genes encoding proinflammatory cytokines and chemokines.

Our findings highlight the IL-17 pathway as a potential regulator of musculoskeletal inflammation in alphavirus-induced disease. Currently, the IL-17 pathway is the target of two FDA-approved drugs, brodalumab and secukinumab, which bind IL-17RA and soluble IL-17A, respectively [20–23]. Both are used to treat patients with RA, PsA and psoriasis, and have been shown to alleviate arthritic symptoms and reduce inflammation of the joints. Targeting the IL-17 signaling pathway using FDA-approved drugs could be a valuable therapeutic strategy, either alone or in combination with other complementary immunotherapeutic approaches shown to be effective in experimental models of alphavirus-induced disease.

## Materials and methods

### Ethics statement

For human studies, written, informed consent was obtained from all subjects. The study was approved by the Human Research Ethics Committee of the University of New South Wales (No. 04257). Serum from healthy individuals was provided by Australian Red Cross with written and oral informed consent, approved by Griffith University Human Research Ethics Committee (BDD/01/12/HREC). All animal experiments were conducted in accordance with the “Australian Code for the Care and Use of Animals for Scientific Purposes” as defined by the National Health and Medical Research Council of Australia. RRV work was approved by Griffith University’s Animal Ethics Committee (AEC GLY/14/16, GLY/09/18).

### Virus

Stocks of the T48 strain of RRV (RRV_T48_) were generated from the full-length T48 cDNA clone [123], and propagated in Vero cells. RRV titers were determined by plaque assay on Vero cells as described previously [124].

### Viral plaque assays

Whole feet and quadriceps muscle tissue homogenates and serum were serially diluted (10^1^ to 10^3^), added to Vero cell monolayers and incubated for 1 h at 37°C. Cells were overlaid with DMEM media containing 2% FCS and 1% agarose and incubated for 48 hrs at 37°C. Cells were fixed using 1% formaldehyde and viral plaques stained using 0.1% crystal violet. Serum viral load was expressed as plaque forming units (PFU) per mL. Tissue viral titers were normalised to tissue weight and expressed as PFU/per gram (PFU/g) of tissue. Limit of detection of the assay was 50 PFU/mL.

### Mice, viral infections and *in vivo* monoclonal antibody treatment

C57BL/6J (Animal Resources Centre, Perth, Australia) and IL-17^GFP^ (C57BL/6-*Il17a*^*tm1Bcgen*^/J) mice were used in this study. For RRV infections, 21-day-old male and female mice, (of equal gender distribution in each group) were inoculated subcutaneously in the thorax below the right forelimb with 10^4^ PFU of RRV_T48_ diluted in sterile PBS in a 50 μL volume, and monitored daily. Mock-infected mice were inoculated with 50 μl sterile PBS. Mice were weighed and scored for disease signs every 24 h, as described previously [58]. For *in vivo* neutralisation experiments, mice were injected intraperitoneally with 2.86 mg/kg anti-IL-17A/F monoclonal antibody (clone MA5-23748; Thermo Fisher) or with Rat IgG2a isotype control (clone MAB006; RnD Systems) from 0, 2, 4 days post-infection (dpi) and daily from 6 dpi until 9 dpi. Antibodies were diluted in sterile PBS and administered in a volume of 200 μL.

### Human studies

Serum from RRV-infected individuals was obtained from 6 Caucasian subjects (5 male, 1 female; average age of 44.5 years old) enrolled in the Dubbo Infection Outcomes Study (DIOS) cohort, following confirmation of acute RRV infection (median 4 days post disease onset) [125].

### Flow cytometry

At days 7, 10, 14 and 30 dpi, mice were euthanised and perfused intracardially with ice-cold PBS, and feet and quadriceps muscles were collected. Tissues were digested with Collagenase IV (2mg/mL; Worthington) and DNase I (5μg/mL; Sigma Aldrich) in RPMI and 10% fetal calf serum (FCS; Thermo Fisher Scientific) for 1 h at 37°C, and following mechanical disaggregation to release cells from tissues, cells were filtered through 70μm and 30μm nylon meshes. T cells were stimulated with phorbol myristate acetate (50ng/mL; Sigma Aldrich), ionomycin (1μM; Sigma Aldrich) in IMDM + 10% FCS (Sigma Aldrich), and brefeldin A (10μg/mL; Sigma Aldrich) for 4 h at 37°C prior to intracellular cytokine staining. Cell suspensions were labelled with fluorochrome-conjugated antibodies against CD3 (17A2; Biolegend), CD4 (GK1.5; Biolegend), CD8 (53–6.2; BD Biosciences), CD45 (30-F11; BD Biosciences), TCRβ (Η57–597; eBiosciences), CD11b (M1/70; BD Biosciences), γδTCR (GL-7; eBiosciences), I-A/I-E (M5/114, eBiosciences), Ly6G (1A8; BD Biosciences), Ly6C (AL-21; BD Biosciences), CD90.2 (53–2.1; BD Biosciences), ICAM-1 (3E2; BD Biosciences), VCAM-1 (429; BD Biosciences), Podoplanin (8.1.1; BD Biosciences), CD31 (MEC13.3; BD Biosciences), CCR2 (475301; BD Biosciences), CX_3_CR1 (Z8-50; BD Biosciences), CD64 (X54-5/7.1; BD Biosciences), CD103 (2E7; BD Biosciences), KLRG1 (2F1; BD Biosciences), CD69 (H1.2F3; BD Biosciences), and LIVE/DEAD (ThermoFisher Scientific). Intracellular cytokine staining for IL-17A (TC11-18h10, BD Biosciences), IL-17F (REA666, Miltenyi) and IFNγ (XMG1.2; BD Biosciences) was performed using a Cytofix/Cytopermkit (BD Biosciences), according to manufacturer’s instructions. Sphero Blank Calibration Particles (BD Biosciences) were used as counting beads. Cells were acquired on a LSR II Fortessa flow cytometer (BD Biosciences) and gating strategy described in Fig 3A was used to detect tissue-infiltrating cell subsets. Data was analysed using FlowJo software (v10.6; Treestar, Inc.). Clustering analysis was performed on concatenated FCS3.1 files that were downsampled using Downsample v3 plugin (FlowJo V10.8), and the clustering was performed using FlowSOM [126]. Dimensionality reduction analysis done using the UMAP plugin [127], and clusters were visualised using ClusterExplorer (v 1.6.3).

### Cell sorting and total RNA extraction

Mice were infected with RRV_T48_ and at 10 days post-infection, mice were perfused intracardially with ice-cold PBS, and feet were collected and digested with Collagenase IV (2mg/mL; Worthington) and DNase I (5μg/mL; Sigma Aldrich) in RPMI and 10% fetal calf serum (FCS; Thermo Fisher Scientific) for 1 h at 37°C. Following mechanical disaggregation to release cells from tissues, single-cell suspensions were filtered through 70um filters and immunolabelled using antibodies against mouse CD45 (30-F11; BD Biosciences, CD11b (M1/70; BD Biosciences), Ly6G (1A8; BD Biosciences), Ly6C (HK1.4; Biolegend), CD3 (17A2; Biolegend), CD31 (MEC 13.3; BD Biosciences). Dead cells were excluded using LIVE/DEAD NIR (M5/114, eBiosciences). CD45^+^ and CD45^-^ cells were sorted on a FACS Aria Fusion (BD Biosciences) in sorting buffer (RPMI 10% FCS; Thermo Fisher Scientific, 5 mM EDTA; Sigma Aldrich) using gating strategy described in S1 Fig. Cells were sorted to a purity of 99% and collected in RNase-free Eppendorf tubes. Total RNA was isolated from sorted cell pellets using a Maxwell simplyRNA Tissue kit (Promega, USA) on the Maxwell 48 RSC automated nucleic acid purification platform, according to manufacturer’s instructions. RNA concentration was assessed using RNA QuantiFluor RNA system (Promega, USA).

### Immunofluorescence staining and confocal microscopy

Quadriceps muscles were fixed in periodate-lysine paraformaldehyde (PLP) buffer (0.1M L-Lysine, 0.2M phosphate buffer, NaIO_4_, 4% PFA), and whole feet fixed in 4% PFA for 48 h, and dehydrated overnight in 30% sucrose (in PBS w/v), embedded in OCT (TissueTek; Sakura) and snap-frozen. Feet were decalcified in 14% EDTA prior to embedding. Coronal and transverse sections of quadriceps and foot tissue (respectively) between 30μm—100μm–thick were cut on a cryomicrotome (Leica 1860UV), permeabilised (0.05% Triton X-100; Sigma Aldrich), 2% FCS (v/v in PBS)), blocked (DAKO, X0909), and stained with Hoechst 33258 or diaminido-2-phenylindol (DAPI; ThermoFisher Scientific), Phalloidin AlexaFluor 594 (ThermoFisher Scientific) and antibodies against mouse CD4 (RM4-5, BD Biosciences), CD8 (53–6.7; BD Biosciences), Collagen Type IV (ab19808; Abcam), Ly6G AlexaFluor 647 (BD Biosciences; 1A8), IA/IE (MHC-II, M5/114, BD Biosciences), Podoplanin (gp38, 8.1.1; Biolegend). Primary antibodies were detected using anti-rat or anti-rabbit AlexaFluor 488, AlexaFluor 568, AlexaFluor 647 (ThermoFisher Scientific) or Streptavidin-conjugated eFluor570 (eBiosciences) or Streptavidin DyLight649 (ThermoFisher). Sections stained with biotinylated antibodies were first blocked using an Avidin/Biotin blocking kit (DAKO) for 15 min each before staining. Transverse vibratome sections of decalcified feet were cut using a vibratome (Leica VT1200S), permeabilised and immunolabelled with antibodies and cleared using the clearing-enhanced 3D (Ce3D) tissue clearing protocol [128] or using RapiClear RC1.49 (Sunjin Labs) according to an optimised protocol. Slides were mounted either in Prolong Gold Antifade (ThermoFisher Scientific) or in refractive index-matching solution (RIMS) and z-stack images were acquired at a 1024x1024 pixel resolution with a 20X/0.8 NA or a silicone-immersion 30XS/1.25NA objective on an Olympus FV3000 confocal microscope. Raw images were processed and analysed using Imaris (9.0.2; Bitplane). Figures and movies were prepared using Adobe Illustrator and Adobe After Effects, respectively.

### Histological staining and analysis

Mice were euthanised and perfused intracardially with ice-cold PBS. Quadriceps muscle and knee tissues were fixed in 4% paraformaldehyde (PFA) and embedded in paraffin. Knees were decalcified in 14% EDTA prior to embedding. Five micrometer–thick coronal sections of the knee, and transverse sections of quadriceps muscle were stained with hematoxylin and eosin (H&E). Images were acquired using a 20X objective BX53 microscope (Olympus) and analysed using CellSens software (Olympus). For automated quantification of nuclei, H&E–stained slides were scanned on an automated slide scanner (Aperio AT; Leica) and images analysed using Aperio ImageScope software (v12; Leica) and nuclei counted using the Nuclear v9 algorithm. Five micrometer–thick transverse knee sections were stained with safranin O. Cartilage thickness and damage was assessed from the medial and lateral femoral condyle and medial and lateral tibial condyle using a modified version of semiquantitative scoring system of Glasson et al [65]. Scores were based on loss of safranin O staining as a measure of cartilage proteoglycan depletion, where *0*: normal cartilage, no depletion of safranin O stain; *1*: mild loss of safranin O stain; *2*: moderate loss of safranin O stain; *3*: moderate to severe loss of safranin O stain, thinning of cartilage; *4*: complete loss of safranin O stain, thinning of cartilage. Sections were scored in a blinded manner by five independent observers.

### Quantification of tissue cytokines

Whole feet, quadriceps muscles and knees were homogenized in cold, sterile PBS and supernatants used to detect soluble tissue protein. Protein quantification was performed using a mouse Inflammation Cytometric Bead Array (CBA) kit (BD Biosciences), according to manufacturer’s instructions, and detected using an LSR II Fortessa flow cytometer (BD Biosciences). Protein levels were analysed using FCAP Array software (v 3.0) (BD Biosciences) and normalised to tissue weight (g) and expressed as pg per gram of tissue (pg/g) or pg/mL for serum. Assay limit of detection was 20pg/mL. Quantification of IL-17 protein in human sera from RRV patients was performed using a human IL-17A Quantikine HS ELISA kit (R&D Systems) according to manufacturer’s instructions. Assay limit of detection was 0.2pg/mL.

### Quantification of mRNA expression in tissues

Whole feet and quadriceps muscles were homogenized in TRIzol (Invitrogen) to extract total RNA, according to manufacturer’s instructions. Total RNA (1 μg/mL) was reverse transcribed into cDNA using Reverse Transcriptase (Sigma Aldrich) and Oligo(dT)_15_ primer (Promega), and quantitative real-time PCR (qRT-PCR) was performed using IL-17 Quantitect primers (Qiagen, QT00103278). qRT-PCR was performed on a CFX96 Touch Real-Time PCR machine (Bio-Rad Laboratories) using SYBR Green (Sigma Aldrich) and samples run in duplicate. Samples were analysed using CFX Manager software (Bio-Rad Laboratories) and normalized against housekeeping gene (*Gapdh*) and expressed as relative fold-change in expression relative to *Gapdh* in mock-infected tissue using the 2^-ΔΔCt^ method [129]. To assess RRV viral load, cDNA was obtained from tissue homogenates as described above, with the exception of using random nonamers (Sigma Aldrich) in place of Oligo(dT)_15_ primer. qRT-PCR was performed using 1μg of cDNA and SsoAdvanced Universal Probes Supermix (Bio-Rad Laboratories). Viral load was assessed by detection of non-structural protein 3 (*nsP3—*Forward primer: 5’-CCGTGGCGGGTATTTATCAAT-3’; Reverse primer: 5’-AACACTCCCGTCGACAACAGA-3’) region primers (Sigma Aldrich) and RRV T_48_ probe (Sigma Aldrich; 5’-(6FAM)ATTAAGAGTGAGC-3’). Primers for mouse *Ifnb1*, *Isg15* and *Irf7* (Sigma Aldrich) were used (Sequences in S1 Table). A standard curve was generated using serial dilutions of RRV T_48_ infectious plasmid ranging from 0 fg to 10 ng of known RRV copy numbers, which was used to interpolate results and calculate number of RRV copies in tissue homogenates (expressed in log_10_/μg of RNA). Assay limit of detection was 0.9fg, or 55 copies of RRV RNA.

### Nanostring differential gene expression analysis

RNA target molecules were quantified using a nCounter mouse Metabolic Pathways panel (catalog no. LBL-10726-01; Nanostring Technologies), which includes 748 genes involved in the core pathways and processes that define immune and cellular metabolism, and 20 internal reference genes for data normalization. Samples were processed according to the nCounter gene expression protocol. Briefly, 200 ng of RNA (in a 5 μl volume), 8 μl of Master Mix (Hybridization buffer and Reporter CodeSet) and 2 μl of Capture ProbeSet were used for hybridization. After 20 h hybridization at 65°C, excess probes were washed using a two-step magnetic bead-based purification system on an nCounter Prep-station instrument (Nanostring, USA) and immobilized in a sample cartridge for data collection. Data collection was performed on the nCounter digital analyzer (Nanostring, USA) with a field-of-view FOV555. Data was analyzed by ROSALIND (https://rosalind.onramp.bio/) following the Nanostring nCounter Advanced Analysis protocol of dividing counts within a lane by the geometric mean of the normalizer probes from the same lane; housekeeping probes used for normalization were selected based on the geNorm algorithm as implemented in the NormqPCR R library. Fold changes and *p* values were calculated using the Optimal method as described in the nCounter Advanced Analysis 2.0 User Manual. P-value adjustment was performed using the Benjamini-Hochberg method of estimating false discovery rates (FDR). Genes with a differential expression at *p*<0.05 were clustered for visual analysis using unsupervised hierarchical clustering using ROSALIND software. Statistical significance was set at *p* < 0.05. Volcano plots were generated using VolcaNoseR [130] and heatmaps of unsupervised hierarchical clustering of rows and columns were generated based on *z*-scores (Euclidian distance) using Morpheus (https://software.broadinstitute.org/morpheus/).

### Statistical analysis

Statistical analyses were performed using GraphPad Prism v9.0 software (GraphPad Software Inc., San Diego, California). Differences between groups were analysed using a Mann-Whitney U test and results shown as a mean +/- SEM and 95% confidence intervals (CI). For semiquantitative cartilage damage scores using safranin O, results were expressed as a median +/- SEM. Differences were considered statistically significant when *p < 0*.*05*.

## Supporting information

S1 Fig Population frequencies of T cells isolated from the feet of RRV-infected mice. Percentage of T cell populations (relative to parent populations) corresponding to total cell counts in Fig 3. **A)** Percentages (of parent CD45^+^ Lin- populations) of CD3^+^, CD4^+^ and CD8^+^ T cells in the feet, **B)** muscle of RRV-infected IL-17^GFP^ mice at 7, 10 and 14 dpi. C) Percentages (of parent CD4^+^ or CD8^+^ populations) of IL-17A^+^ T cells in the feet and **B)** muscle of RRV-infected IL-17^GFP^ mice at 7, 10 and 14 dpi. E) Percentage (of CD45^+^Lin-CD3^+^ populations) of γδTCR^+^ T cells in the feet. Data representative of 3 independent experiments (n = 5 mice per group) and statistically significant differences between groups for each time point determined by Mann-Whitney U Test. *, *p* < 0.05; **, *p* < 0.01; *** *p<0*.*005*; **** *p<0*.*001*; ns: not significant.
(TIF)

S2 Fig Enumeration of CD3^+^ T cells in the feet of RRV-infected mice at Day 30 post-infection. Total CD3^+^ and CD8^+^ and CD4^+^ T cells expressing tissue-resident memory T cell (T_RM_) marker CD103 and KLRG1 were detected by flow cytometry in RRV-infected IL-17^GFP^ mice. Statistical differences were assessed by a Mann-Whitney *U* Test. Significant differences shown on the graph (*p < 0.*05*; ns = not significant).
(TIF)

S3 Fig Confocal immunofluorescence microscopy of foot tissue in RRV-infected IL-17^GFP^ mice at 10 dpi. Thick cryosections of feet from RRV-infected IL-17^GFP^ mice were immunolabelled for MHC-II, Podoplanin and CD3. Images shown of low (upper panel) and high (lower panel) magnification of areas with MHC-II^+ve^ co-localising with IL-17^GFP^ signal. Arrows in maximum intensity project (middle panels) and 3D-rendered surface volumes (right panels) show co-localized signal. Confocal mages acquired as *z*-stacks; scale bars shown in individual panels.
(TIFF)

S4 Fig Differential expression of Type I interferon-associated genes in RRV-infected mice. mRNA expression of A) IRF7 and B) ISG15 was measured in the feet and muscle of RRV-infected C57BL/6J mice treated with IL-17A/F mAb or IgG2a isotype. Tissues were collected at 3 **(A)** and 3 and 10 dpi **(B)** and relative mRNA expression of IRF7 and ISG15 was measured, respectively. Expressed as relative fold-change in mRNA expression normalised against housekeeping gene relative to mock-infected tissue Data are presented as the mean +/- SEM; n = 5 mice per group. Statistically significant differences between groups were determined with Mann-Whitney U test, *p* values indicated on graphs. ns: not significant.
(TIF)

S5 Fig Expression of CCR2 and CX_3_CR1 by synovial inflammatory monocytes and macrophages. **A)** Representative flow cytometry plots of synovial monocytes (LIVE CD45^+^ Ly6G^-^ CD11b^+^ Ly6C^hi^) and macrophages (LIVE CD45^+^ Ly6G^-^ CD11b^+^ Ly6C^int/lo^) isolated from the feet of RRV-infected C57BL/6J mice treated with IL-17A/F mAb or IgG2a isotype at 10 dpi. CD11b^+^ Ly6C^hi^ inflammatory monocytes gated on CCR2^+^ CX_3_CR1^+^ subsets and CD11b^+^ Ly6C^lo/int^ macrophages gated on CD64^+^ CX_3_CR1^+^ subsets. Gates show frequency of parent population. Frequency of parent populations shown for **(B)** CCR2^+^ CX_3_CR1^+^ inflammatory monocytes and **(C)** CD64^+^ CX_3_CR1^+^ macrophages.
(TIF)

S6 Fig Gating strategy for cell sorting of CD45^+^ and CD45^-^ compartments from the feet of RRV-infected mice. Cells were isolated from the foot joints of RRV-infected C57BL/6J mice treated with IL-17A/F mAb or IgG2a isotype at 10 dpi (as described in Materials and Methods). To sort CD45^+^ and CD45^-^ cell populations, doublet cells were excluded and then gated on live cells. From live cells, CD45^+^ (green gate) and CD45^-^ (blue gate) cell populations were collected in separate tubes. CD45^+^ and CD45^-^ cells were sorted to a purity of 99% and lysed for total RNA extraction for the experiment described in Fig 11.
(TIF)

S1 Table List of primer sequences (referred to in Materials and Methods) for mouse Ifnb1, Isg15 and Irf7. Forward and reverse sequences shown. (TIFF)

S1 Movie Animated video of optically-cleared feet of uninfected and RRV-infected IL-17^GFP^ mice (related to Fig 4A). Decalcified feet of uninfected or RRV-infected mice at 10 dpi, were immunolabelled with anti-Collagen IV antibody, and counterstained with DAPI. 150μm-thick immunolabelled vibratome sections were optically cleared and mounted in Ce3D clearing medium, and imaged by confocal laser scanning microscopy using a 30XS / 1.25NA silicone immersion objective.
(MP4)
