# Correction: Interleukin-17 contributes to Ross River virus-induced arthritis and myositis

**DOI:** 10.1371/journal.ppat.1010519

**Published:** 2022-04-27

**Authors:** The PLOS Pathogens Staff

The last two authors, Suresh Mahalingam and Ali Zaid, should have been noted as joint senior authors. The publisher apologizes for the error.
