# Supplementary figures and images for: Interleukin-17 contributes to Ross River virus-induced arthritis and myositis
Source: PLoS Pathog. 2022 Feb 10;18(2):e1010185. doi: 10.1371/journal.ppat.1010185 (PMC8830676; doi:10.1371/journal.ppat.1010185)

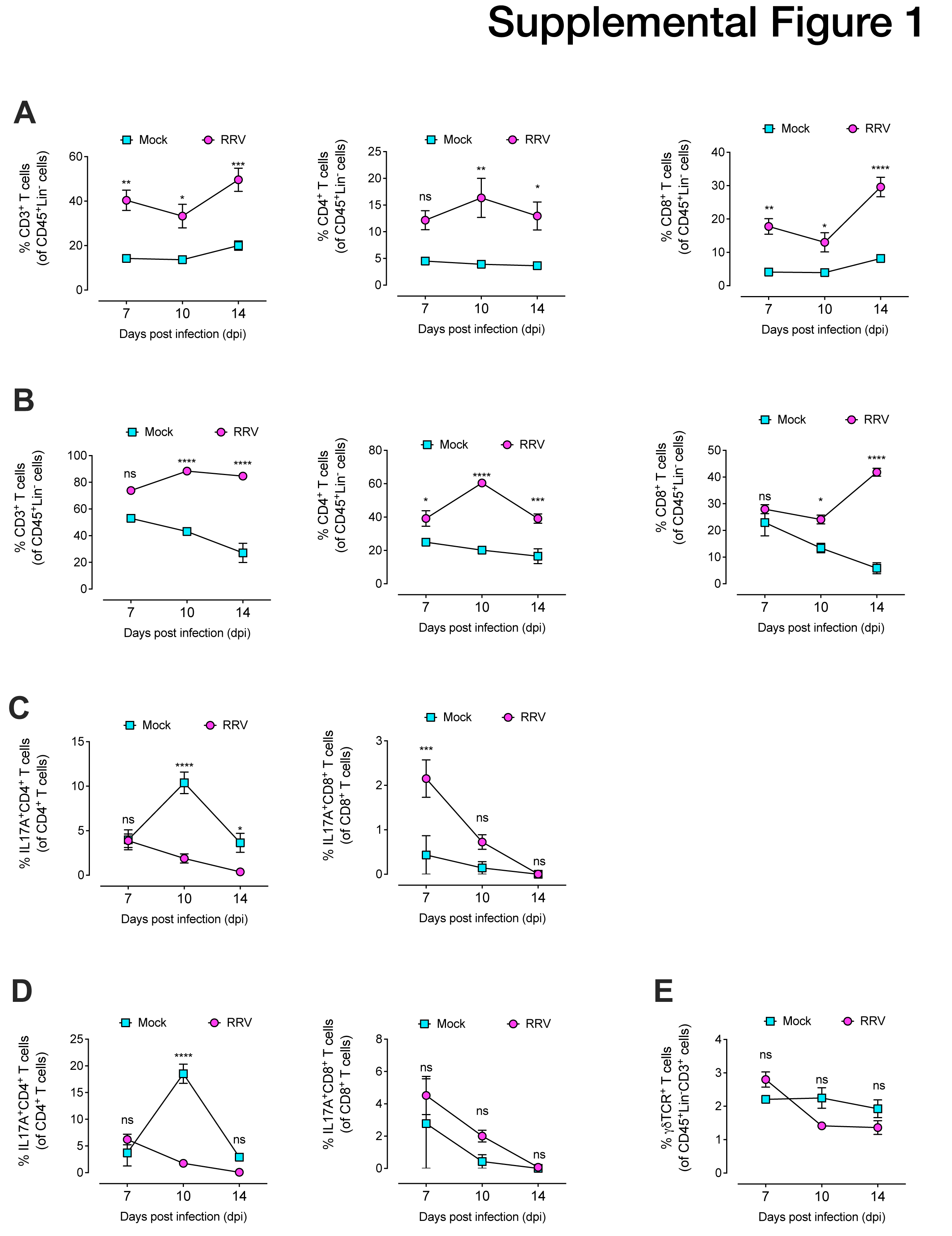

Supplement: S1 Fig — Percentage of T cell populations (relative to parent populations) corresponding to total cell counts in Fig 3. A) Percentages (of parent CD45+ Lin- populations) of CD3+, CD4+ and CD8+ T cells in the feet, B) muscle of RRV-infected IL-17GFP mice at 7, 10 and 14 dpi. C) Percentages (of parent CD4+ or CD8+ populations) of IL-17A+ T cells in the feet and B) muscle of RRV-infected IL-17GFP mice at 7, 10 and 14 dpi. E) Percentage (of CD45+Lin-CD3+ populations) of γδTCR+ T cells in the feet. Data representative of 3 independent experiments (n = 5 mice per group) and statistically significant differences between groups for each time point determined by Mann-Whitney U Test. *, p < 0.05; **, p < 0.01; *** p<0.005; **** p<0.001; ns: not significant. (TIF) [file ppat.1010185.s001.tif]

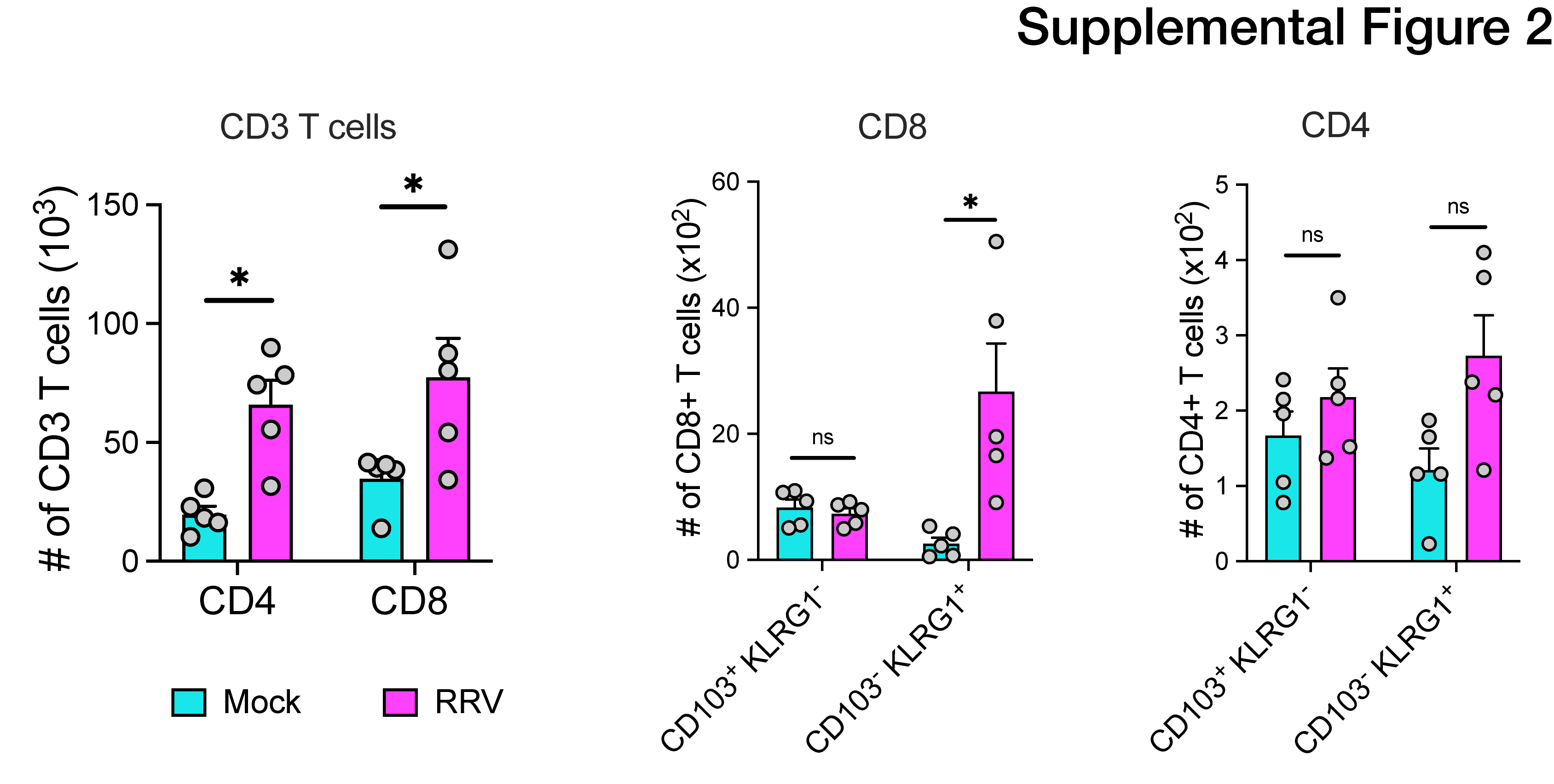

Supplement: S2 Fig — Total CD3+ and CD8+ and CD4+ T cells expressing tissue-resident memory T cell (TRM) marker CD103 and KLRG1 were detected by flow cytometry in RRV-infected IL-17GFP mice. Statistical differences were assessed by a Mann-Whitney U Test. Significant differences shown on the graph (*p < 0.05; ns = not significant). (TIF) [file ppat.1010185.s002.tif]

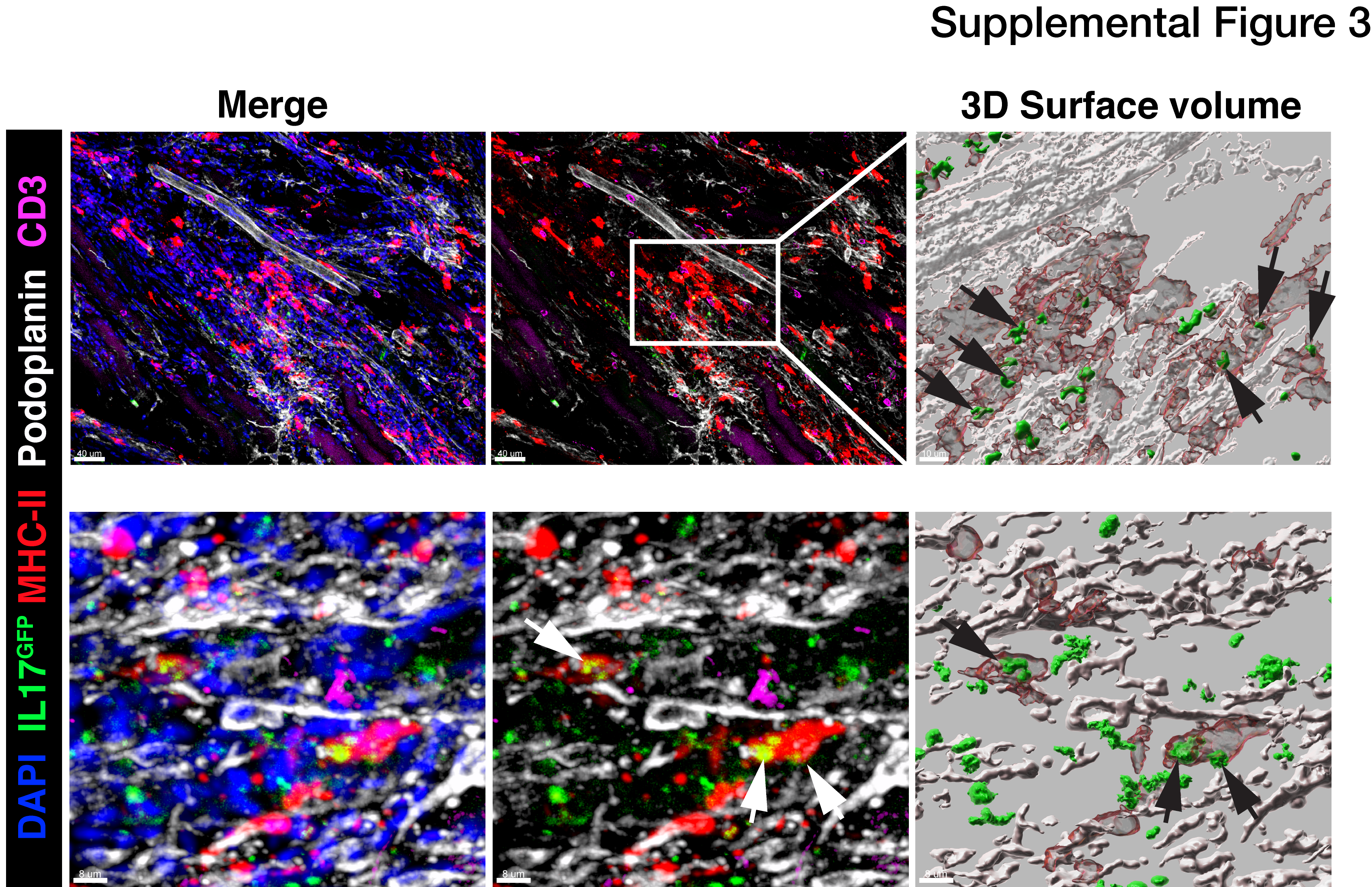

Supplement: S3 Fig — Thick cryosections of feet from RRV-infected IL-17GFP mice were immunolabelled for MHC-II, Podoplanin and CD3. Images shown of low (upper panel) and high (lower panel) magnification of areas with MHC-II+ve co-localising with IL-17GFP signal. Arrows in maximum intensity project (middle panels) and 3D-rendered surface volumes (right panels) show co-localized signal. Confocal mages acquired as z-stacks; scale bars shown in individual panels. (TIFF) [file ppat.1010185.s003.tiff]

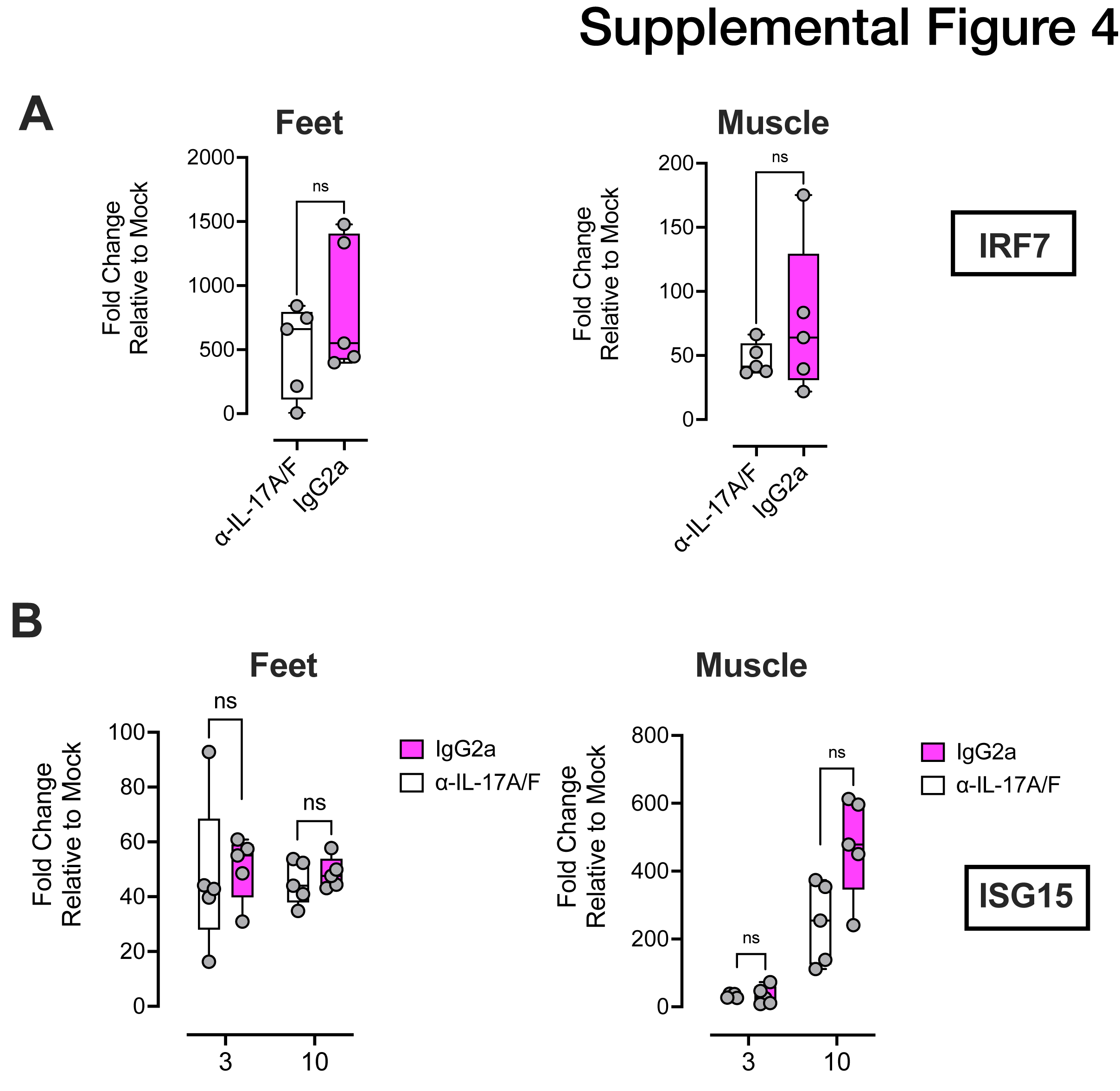

Supplement: S4 Fig — mRNA expression of A) IRF7 and B) ISG15 was measured in the feet and muscle of RRV-infected C57BL/6J mice treated with IL-17A/F mAb or IgG2a isotype. Tissues were collected at 3 (A) and 3 and 10 dpi (B) and relative mRNA expression of IRF7 and ISG15 was measured, respectively. Expressed as relative fold-change in mRNA expression normalised against housekeeping gene relative to mock-infected tissue Data are presented as the mean +/- SEM; n = 5 mice per group. Statistically significant differences between groups were determined with Mann-Whitney U test, p values indicated on graphs. ns: not significant. (TIF) [file ppat.1010185.s004.tif]

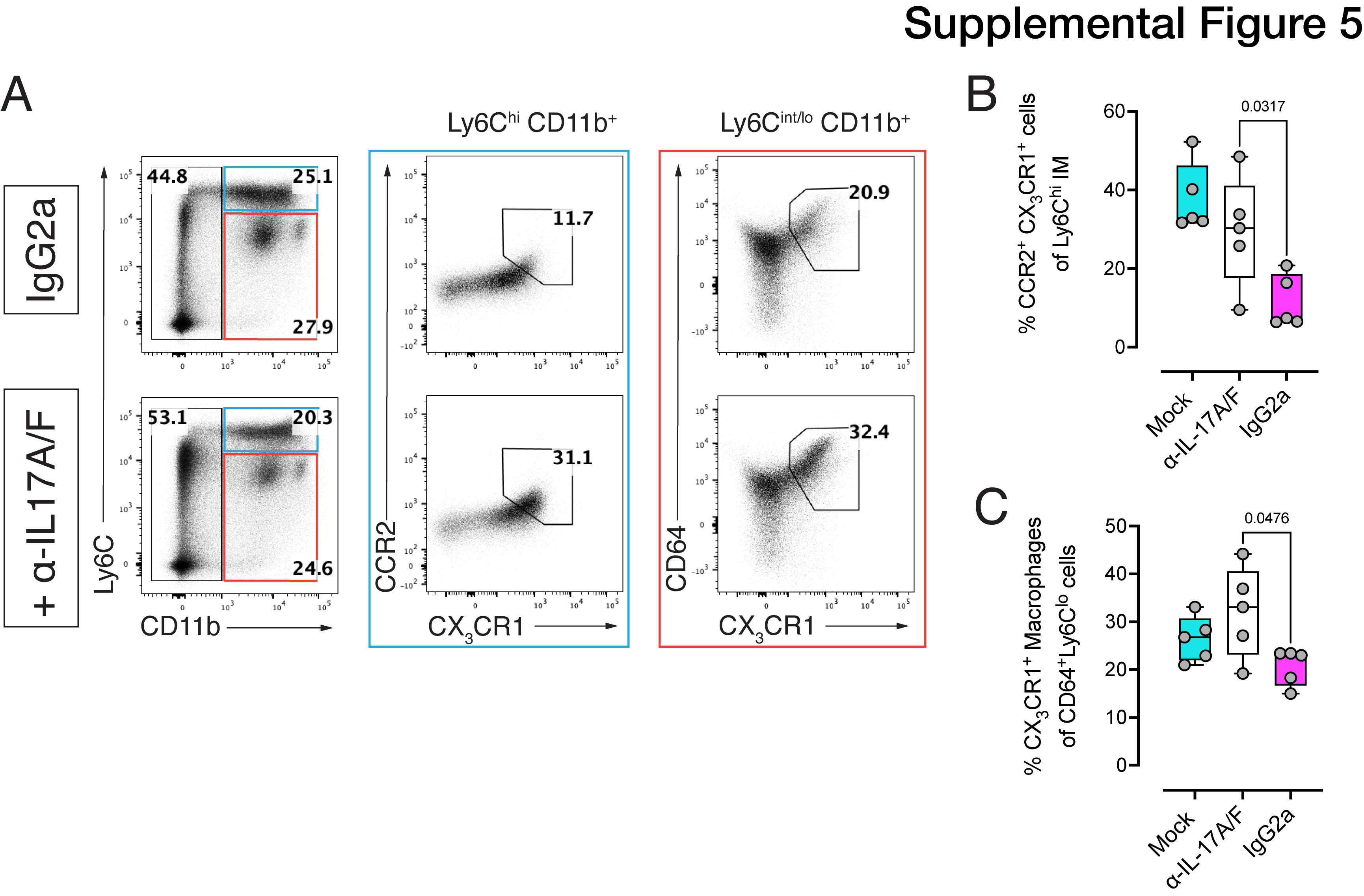

Supplement: S5 Fig — A) Representative flow cytometry plots of synovial monocytes (LIVE CD45+ Ly6G- CD11b+ Ly6Chi) and macrophages (LIVE CD45+ Ly6G- CD11b+ Ly6Cint/lo) isolated from the feet of RRV-infected C57BL/6J mice treated with IL-17A/F mAb or IgG2a isotype at 10 dpi. CD11b+ Ly6Chi inflammatory monocytes gated on CCR2+ CX3CR1+ subsets and CD11b+ Ly6Clo/int macrophages gated on CD64+ CX3CR1+ subsets. Gates show frequency of parent population. Frequency of parent populations shown for (B) CCR2+ CX3CR1+ inflammatory monocytes and (C) CD64+ CX3CR1+ macrophages. (TIF) [file ppat.1010185.s005.tif]

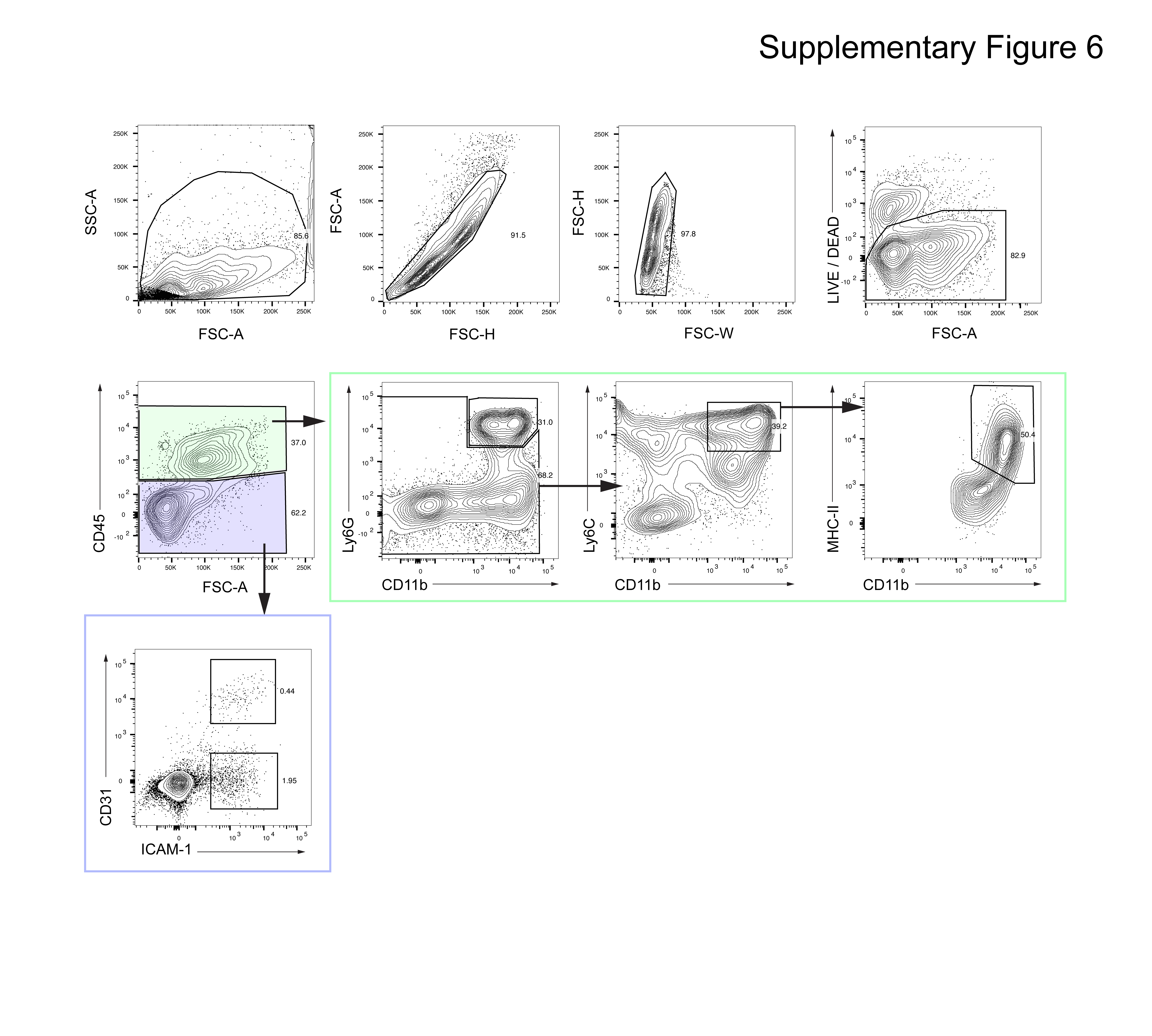

Supplement: S6 Fig — Cells were isolated from the foot joints of RRV-infected C57BL/6J mice treated with IL-17A/F mAb or IgG2a isotype at 10 dpi (as described in Materials and Methods). To sort CD45+ and CD45- cell populations, doublet cells were excluded and then gated on live cells. From live cells, CD45+ (green gate) and CD45- (blue gate) cell populations were collected in separate tubes. CD45+ and CD45- cells were sorted to a purity of 99% and lysed for total RNA extraction for the experiment described in Fig 11. (TIF) [file ppat.1010185.s006.tif]

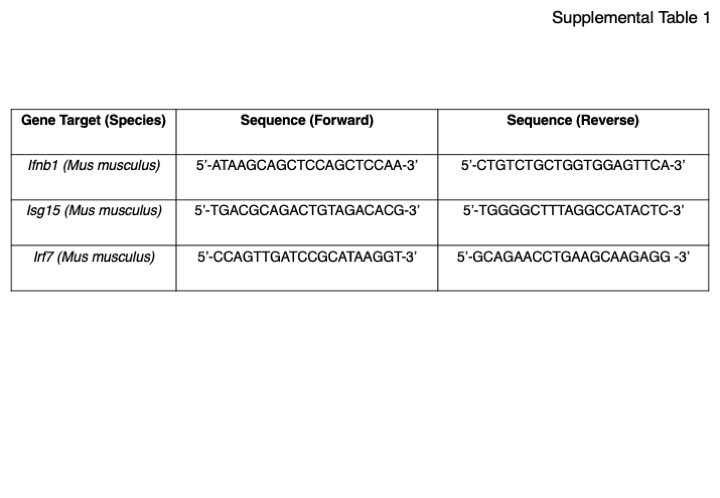

Supplement: S1 Table — (TIFF) [file ppat.1010185.s007.tiff]
